# Transcription inhibition by the depsipeptide antibiotic salinamide A

**DOI:** 10.7554/eLife.02451

**Published:** 2014-04-30

**Authors:** David Degen, Yu Feng, Yu Zhang, Katherine Y Ebright, Yon W Ebright, Matthew Gigliotti, Hanif Vahedian-Movahed, Sukhendu Mandal, Meliza Talaue, Nancy Connell, Eddy Arnold, William Fenical, Richard H Ebright

**Affiliations:** 1 Waksman Institute, Rutgers University, Piscataway, United States; 2 Center for Biodefense, New Jersey Medical School, Rutgers University, Newark, United States; 3 Center for Advanced Biotechnology and Medicine, Rutgers University, Piscataway, United States; 4 Center for Marine Biotechnology and Biomedicine, Scripps Institution of Oceanography, University of California, San Diego, La Jolla, United States; National Institute of Child Health and Human Development, United States

**Keywords:** RNA polymerase, transcription, inhibitor, antibiotic, bridge helix, bridge-helix cap, *E. coli*

## Abstract

We report that bacterial RNA polymerase (RNAP) is the functional cellular target of the depsipeptide antibiotic salinamide A (Sal), and we report that Sal inhibits RNAP through a novel binding site and mechanism. We show that Sal inhibits RNA synthesis in cells and that mutations that confer Sal-resistance map to RNAP genes. We show that Sal interacts with the RNAP active-center ‘bridge-helix cap’ comprising the ‘bridge-helix N-terminal hinge’, ‘F-loop’, and ‘link region’. We show that Sal inhibits nucleotide addition in transcription initiation and elongation. We present a crystal structure that defines interactions between Sal and RNAP and effects of Sal on RNAP conformation. We propose that Sal functions by binding to the RNAP bridge-helix cap and preventing conformational changes of the bridge-helix N-terminal hinge necessary for nucleotide addition. The results provide a target for antibacterial drug discovery and a reagent to probe conformation and function of the bridge-helix N-terminal hinge.

**DOI:** http://dx.doi.org/10.7554/eLife.02451.001

## eLife digest

The need for new antibiotics is becoming increasingly critical, as more and more bacteria become resistant to existing drugs. To develop new treatments, researchers need to understand how antibiotics work. One way antibiotics can kill bacteria is by targeting an enzyme called bacterial RNA polymerase. This enzyme builds chains of RNA that bacteria need to survive.

Sal is an antibiotic produced by a marine bacterium found on the surface of a species of jellyfish. Degen, Feng et al. show that Sal kills bacteria by inhibiting bacterial RNA polymerase and explain how Sal inhibits RNA polymerase. Sal binds to a rod-like structural element within RNA polymerase known as the ‘bridge helix’. The bridge helix has been proposed by others to contain two ‘hinges’ that open and close—allowing the bridge helix to bend and unbend—at specific steps in the cycle through which RNA polymerase builds an RNA chain. Degen, Feng et al. show that Sal binds directly to one of the two hinges and show that Sal binds to the hinge in the unbent state. Therefore, Degen, Feng et al. propose that Sal inhibits the enzyme by preventing the hinge from bending.

The binding site on RNA polymerase for Sal is different from, and does not overlap, the binding sites of current antibacterial drugs. As a result, Sal is able to kill bacteria that are resistant to current antibacterial drugs. When Degen, Feng et al. administered Sal in combination with a current antibacterial drug that targets RNA polymerase, bacteria did not detectably develop resistance to either Sal or the current antibacterial drug.

The structure of the complex between Sal and RNA polymerase suggests several ways that Sal could be modified to improve its ability to interact with RNA polymerase, thereby potentially increasing Sal's antibacterial activity. Future research could develop a range of new drugs based on Sal that could kill bacteria more effectively.

**DOI:** http://dx.doi.org/10.7554/eLife.02451.002

## Introduction

**Figure 1.**
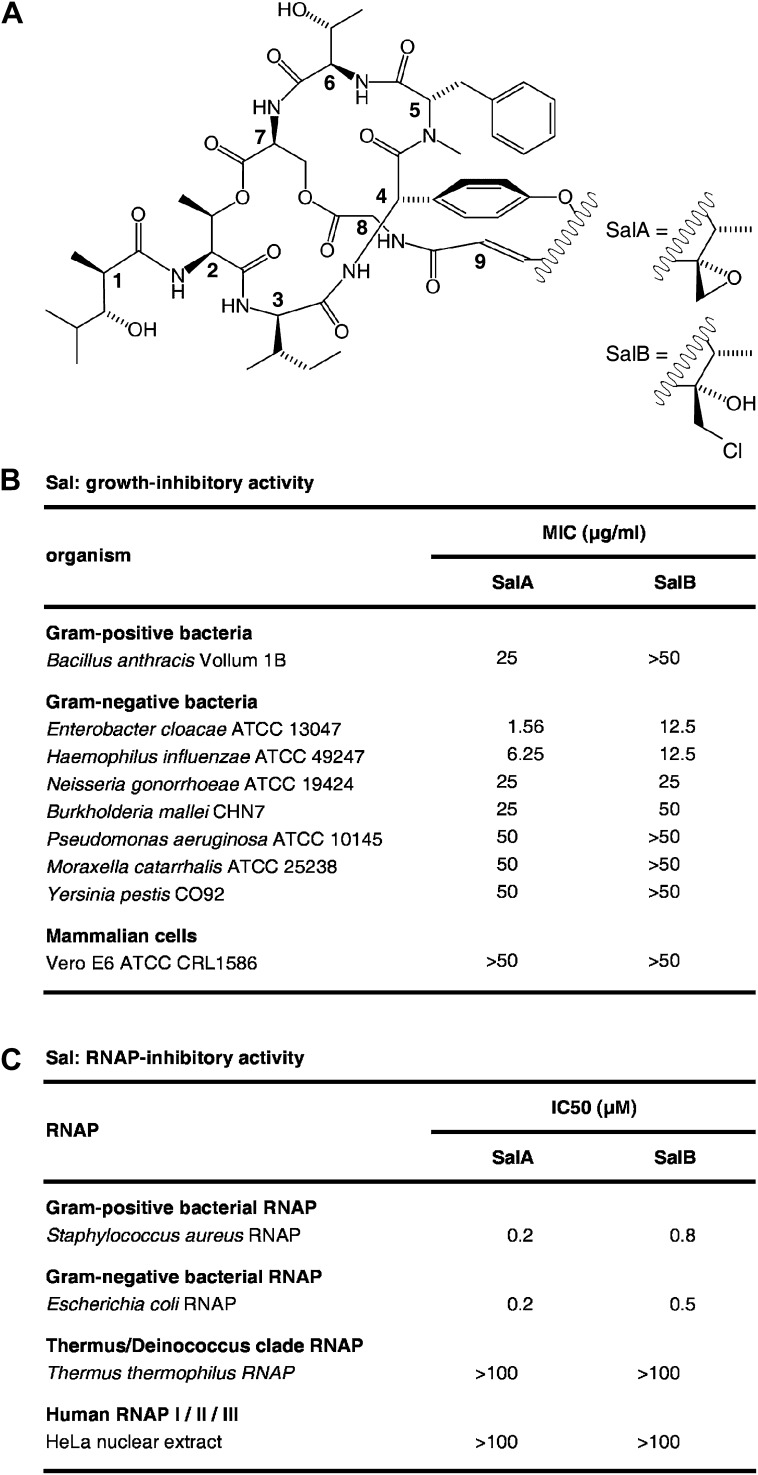
Sal. (**A**) Structures of SalA and SalB (Moore et al., 1999). (**B**) Growth-inhibitory activity of SalA and SalB. (**C**) RNAP-inhibitory activity of SalA and SalB. **DOI:** http://dx.doi.org/10.7554/eLife.02451.003

Salinamide A (Sal; SalA) and salinamide B (SalB) are structurally related bicyclic depsipeptide antibiotics, each consisting of seven amino acids and two non-amino-acid residues (Trischman et al., 1994; Moore et al., 1999; Figure 1A). SalA and SalB are produced by *Streptomyces* sp. CNB-091, a marine bacterium isolated from the surface of the jellyfish *Cassiopeia xamachana* (Trischman et al., 1994; Moore and Seng, 1998; Moore et al., 1999), and SalA also is produced by *Streptomyces* sp. NRRL 21611, a soil bacterium (Miao et al., 1997). SalA and SalB exhibit antibacterial activity against both Gram-positive and Gram-negative bacterial pathogens, particularly *Enterobacter cloacae* and *Haemophilus influenzae*, but do not exhibit cytotoxicity against mammalian cells (Trischman et al., 1994; Moore et al., 1999; Figure 1B). SalA and SalB inhibit both Gram-positive and Gram-negative bacterial RNA polymerase (RNAP) in vitro, but do not inhibit human RNAP I, II, or III in vitro (Miao et al., 1997; Figure 1C). A total synthesis of SalA has been reported (Tan and Ma, 2008).

Although previous work had established that Sal exhibits RNAP-inhibitory activity in a purified system in vitro and antibacterial activity in culture (Trischman et al., 1994; Miao et al., 1997; Moore et al., 1999), previous work had not established a causal relationship between the RNAP-inhibitory activity of Sal and the antibacterial activity of Sal (i.e., had not established that RNAP is the functional cellular target of Sal). In addition, previous work had not provided information regarding the binding site, mechanism, and structural basis of inhibition of RNAP by Sal.

In this work, we show that RNAP is the functional cellular target of Sal, we show that Sal inhibits RNAP through a novel binding site and novel mechanism, we determine crystal structures that define RNAP–Sal interactions, and we set the stage for structure-based design and semi-synthesis of Sal analogs with improved properties.

## Results

### Sal inhibits RNAP in cells

**Figure 2.**
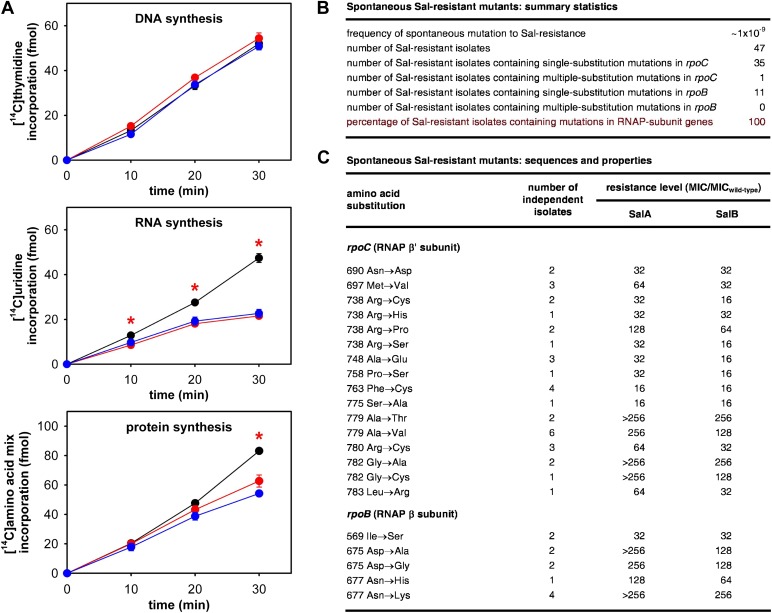
The RNAP-inhibitory activity of Sal accounts for the antibacterial activity of Sal. (**A**) Sal inhibits RNAP in cells. Black, no inhibitor. Red, Sal (2 x MIC). Blue, Rif (2 x MIC). Asterisks, statistically significant differences between no-inhibitor data and Sal data (*t* test; p<0.01). (**B** and **C**) Sal-resistant mutations occur in RNAP subunit genes. MIC_wild-type,SalA_ = 0.049 µg/ml; MIC_wild−type,SalB_ = 0.20 µg/ml. **DOI:** http://dx.doi.org/10.7554/eLife.02451.004

As a first step to determine whether the RNAP-inhibitory activity of Sal is responsible for the antibacterial activity of Sal in culture, we assessed whether Sal inhibits RNAP in bacterial cells in culture. To do this, we assayed macromolecular synthesis by bacterial cells in culture, monitoring incorporation of [^14^C]-thymidine into DNA, [^14^C]-uracil into RNA, and [^14^C]-amino acids into protein. The results in Figure 2A shows that addition of Sal to cultures inhibits RNA synthesis at the first time point following addition and inhibits protein synthesis at later time points. Addition of Sal has no effect on DNA synthesis. The pattern observed for Sal matches the pattern observed for the reference RNAP inhibitor rifampin (Rif; compare red lines and blue lines in Figure 2A; Lancini and Sartori, 1968; Lancini et al., 1969), and matches the pattern expected from first principles for an RNAP inhibitor (i.e., immediate inhibition of RNAP-dependent RNA synthesis and later inhibition of RNA-dependent protein synthesis; Sergio et al., 1975; Irschik et al., 1983, 1985, 1995). We conclude that Sal inhibits RNA synthesis in bacterial cells in culture, and we infer that Sal inhibits RNAP in bacterial cells in culture.

### Sal-resistant mutations occur in RNAP subunit genes

As a second step to determine whether the RNAP-inhibitory activity of Sal is responsible for the antibacterial activity of Sal, we assessed whether Sal-resistant mutations occur in RNAP subunit genes. To do this, we isolated spontaneous Sal-resistant mutants and then PCR-amplified and sequenced genes for RNAP subunits (Figure 2B,C).

Spontaneous Sal-resistant mutants were isolated by plating *E. coli* strain, D21f2tolC—a strain with cell-envelope defects resulting in increased uptake and decreased efflux of small molecules, including Sal (Fralick and Burns-Keliher, 1994; DD and RHE, unpublished)—on agar containing Sal and identifying Sal-resistant colonies. For each Sal-resistant isolate, genomic DNA was prepared and the genes for the largest and second-largest RNAP subunits, *rpoC* encoding RNAP β′ subunit and *rpoB* encoding RNAP β subunit, were PCR-amplified and sequenced. Spontaneous Sal-resistant mutants were isolated with a frequency of ∼1 × 10^−9^ (Figure 2B). A total of 47 independent Sal-resistant mutants were isolated, PCR-amplified, and sequenced (Figure 2B). Strikingly, 100% (47/47) of the analyzed Sal-resistant mutants were found to contain mutations in genes for RNAP subunits: 36 were found to contain mutations in *rpoC* and 11 were found to contain mutations in *rpoB* (Figure 2B).

A total of 21 different substitutions conferring Sal-resistance were identified (Figure 2C). Substitutions were obtained at 11 sites in RNAP β′ subunit (residues 690, 697, 738, 748, 758, 763, 775, 779, 780, 782, and 783) and three sites in RNAP β subunit (residues 569, 675, and 677) (Figure 2C). Quantitation of resistance levels indicated that all mutants exhibited at least moderate-level (≥16-fold) resistance to SalA and SalB, and that nine mutants exhibited high-level (≥128-fold) resistance to SalA (Figure 2C).

In parallel work, we isolated and sequenced induced Sal-resistant mutants (Supplementary file 1). Random mutagenesis of plasmid-borne *rpoC* and *rpoB* genes was performed using error-prone PCR, mutagenized plasmid DNA was introduced into *E. coli* strain D21f2tolC by transformation, transformants were plated on media containing Sal, and Sal-resistant clones were isolated. The plasmid-borne, induced Sal-resistant mutants were found to contain mutations in the same *rpoC* and *rpoB* segments as the spontaneous Sal-resistant mutants (compare Supplementary file 1 and Figure 2C). Transfer of plasmids carrying plasmid-borne, induced Sal-resistant mutants was found to transfer the Sal-resistant phenotype, indicating that no mutation outside of *rpoC* or *rpoB* is required for Sal-resistance.

From the analysis of spontaneous and induced Sal-resistant mutants, we conclude that a single substitution in an RNAP subunit gene, either *rpoC* or *rpoB*, is sufficient to confer Sal-resistance, and we infer that RNAP is the functional cellular target for Sal.

### Sal-resistant mutations define the Sal target

**Figure 3.**
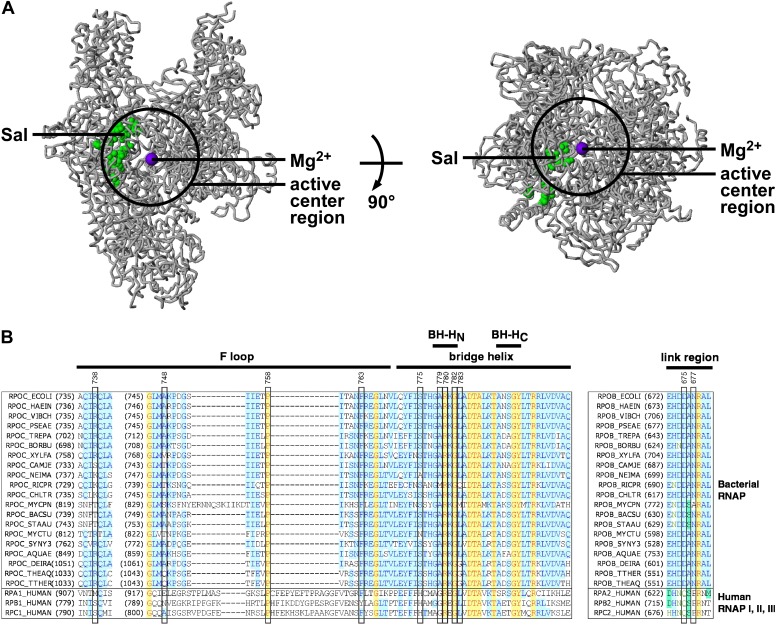
Target of transcription inhibition by Sal. (**A**) The Sal target overlaps the RNAP active-center region. Structure of bacterial RNAP (gray ribbons; black circle for active-center region; violet sphere for active-center Mg^2+^; β' non-conserved region and σ omitted for clarity; PDB 1IW7), showing the sites of Sal-resistant substitutions (green surface; sequences from Figure 2C; ‘Sal target’). Two orthogonal views. (**B**) The Sal target overlaps the RNAP active-center module designated as the ‘bridge-helix cap’ (i.e., the module comprising the N-terminal half of the bridge helix, the F loop, and the link region; Weinzierl, 2010; Hein and Landick, 2010). Sequence alignments of the largest subunits of bacterial RNAP (top 20 sequences) and human RNAP I, RNAP II, and RNAP III (bottom three sequences), showing locations of Sal-resistant substitutions (black rectangles; sequences from Figure 2C; ‘Sal target’), and locations of the RNAP active-center bridge helix, bridge-helix N-terminal hinge (BH-H_C_), bridge-helix C-terminal hinge (BH-H_C_), F loop, and link region (black bars; boundaries from Weinzierl, 2010). **DOI:** http://dx.doi.org/10.7554/eLife.02451.005

In the three-dimensional structure of RNAP, the sites of substitutions conferring Sal-resistance form a tight cluster (‘the Sal target’; green surface in Figure 3A). The dimensions of the Sal target are ∼35 Å × ∼18 Å × ∼12 Å. The Sal target is sufficiently large to be able to encompass Sal (∼16 Å × ∼12 Å × ∼10 Å). Based on the observation that substitutions of the Sal target result in Sal-resistance (Figure 3A), we infer that the Sal target is the binding site for Sal on RNAP.

### The Sal target overlaps the RNAP active-center region

The Sal target is located adjacent to, and partly overlaps, the RNAP active-center region (Figure 3A). We infer that Sal likely inhibits RNAP by inhibiting RNAP active-center function.

Mapping of substitutions conferring Sal-resistance onto the three-dimensional structure of a transcription elongation complex comprising RNAP, DNA, RNA, and an NTP (Vassylyev et al., 2007b) indicates that the Sal target does not overlap the RNAP active-center catalytic Mg^2+^ ion and does not overlap the RNAP residues that interact with the DNA template, the RNA product, and the NTP substrate. We infer that Sal likely inhibits RNAP active-center function allosterically, through effects on RNAP conformation, and not through direct, steric interactions with the RNAP residues that mediate bond formation, template binding, product binding, or substrate binding.

### The Sal target overlaps the RNAP active-center bridge-helix cap

The Sal target overlaps an RNAP active-center module referred to as the ‘bridge-helix cap’, which, in turn, comprises three active-center subregions: the ‘bridge-helix N-terminal hinge’ (BH-H_N_), the ‘F-loop’, and the ‘link region’ (Figure 3B; active-center subregion nomenclature as in Weinzierl 2010 and Hein and Landick 2010). 18 of the 21 identified substitutions conferring Sal-resistance, and all substitutions conferring high-level Sal-resistance, map to these RNAP active-center subregions (Figure 3B). It recently has been proposed that the BH-H_N_ undergoes conformational changes coupled to, and essential for, the nucleotide-addition cycle in transcription initiation and transcription elongation, and that the F-loop, and possibly the link region, control these conformational changes (Hein and Landick, 2010; Weinzierl, 2010; Kireeva et al., 2012; Nedialkov et al., 2013). Specifically, it has been proposed that the BH-H_N_ segment comprising β′ residues 779–783—a segment that includes the sites of 6 of the 21 identified substitutions conferring Sal-resistance, and 4 of the 9 substitutions conferring high-level Sal-resistance—undergoes a hinge-opening/hinge-closing conformational cycle coupled to the nucleotide-addition cycle (Hein and Landick, 2010; Weinzierl, 2010; Kireeva et al., 2012; Nedialkov et al., 2013). (These proposals are supported by results of mutagenesis studies and molecular-dynamics simulations. However, these proposals have not been definitively established. Crystal structures showing an ‘open’ (unbent) BH-H_N_ conformational state have been reported, but a crystal structure showing a ‘closed’ (bent) BH-H_N_ conformational state has not been reported.) Based on the strong, nearly one-for-one, correspondence between the Sal target and the active-center subregions proposed to mediate and control the BH-H_N_ hinge-opening/hinge-closing conformational cycle, we suggest that Sal inhibits RNAP active-center function by inhibiting the proposed BH-H_N_ hinge-opening and/or hinge-closing.

### The Sal target does not overlap targets of the RNAP inhibitors rifampin, streptolydigin, CBR703, myxopyronin, and lipiarmycin

**Figure 4.**
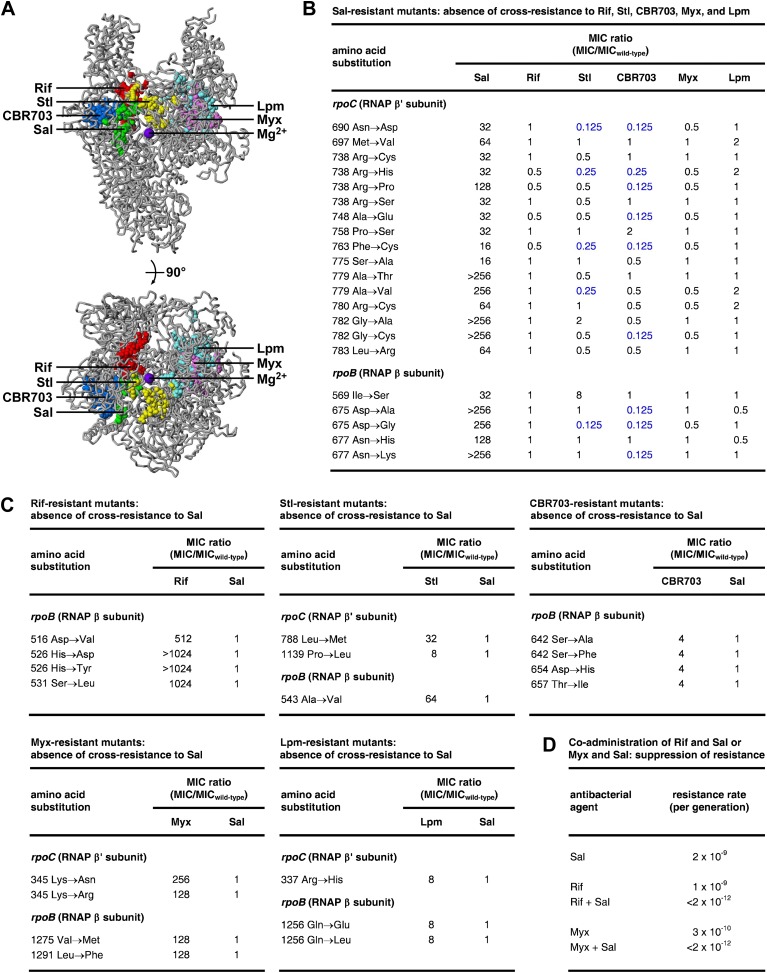
Relationship between the Sal target and the targets of other RNAP inhibitors. (**A**) The Sal target does not overlap the targets of Rif, Stl, CBR703, Myx, and Lpm. Structure of bacterial RNAP (gray ribbons; violet sphere for active-center Mg^2+^; Mukhopadhyay et al., 2008), showing sites of substitutions that confer resistance to Sal (green; Figures 2, 3), Rif (red; Ovchinnikov et al., 1981, 1983; Lisitsyn et al., 1984; Jin and Gross, 1988; Severinov et al., 1993, 1994; Campbell et al., 2001; Garibyan et al., 2003), Stl (yellow; Lisitsyn et al., 1985; Heisler et al., 1993; Severinov et al., 1993, 1995; Tuske et al., 2005), CBR703 (blue; Artsimovitch et al., 2003; X Wang and RHE, unpublished), Myx (magenta; Mukhopadhyay et al., 2008; Srivastava et al., 2011), and Lpm (cyan; Ebright, 2005; Srivastava et al., 2011). Views as in Figure 3. (**B**) Sal-resistant mutants (Figure 2C) do not exhibit cross-resistance to Rif, Stl, CBR703, Myx, and Lpm. Blue, high-level hypersusceptibility (MIC ratio ≤0.25). MIC_wild-type,Sal_ = 0.049 μg/ml; MIC_wild-type,Rif_ = 0.20 μg/ml; MIC_wild-type,Stl_ = 3.13 μg/ml; MIC_wild-type,CBR703_ = 6.25 μg/ml; MIC_wild-type,Myx_ = 0.20 μg/ml; MIC_wild-type,Lpm_ = 1.56 μg/ml. (**C**) Rif-resistant mutants (Jin and Gross, 1988; Garibyan et al., 2003; DD and RHE, unpublished), Stl-resistant mutants (Tuske et al., 2005), CBR703-resistant mutants (Artsimovitch et al., 2003; X Wang and RHE, unpublished), Myx-resistant mutants (Mukhopadhyay et al., 2008), and Lpm-resistant mutants (Ebright, 2005) do not exhibit cross-resistance to Sal. MIC_wild-type,Sal_ = 0.049 μg/ml; MIC_wild-type,Rif_ = 0.20 μg/ml; MIC_wild-type,Stl_ = 1.56 μg/ml; MIC_wild-type,CBR703_ = 6.25 μg/ml; MIC_wild-type,Myx_ = 0.20 μg/ml; MIC_wild-type,Lpm_ = 1.56 μg/ml. (**D**) Co-administration of Sal (2 × MIC) with Rif (2 × MIC) or Myx (2 × MIC) suppresses the emergence of resistance. **DOI:** http://dx.doi.org/10.7554/eLife.02451.006

The Sal target does not overlap the targets of the previously characterized RNAP inhibitors Rif (Ovchinnikov et al., 1981, 1983; Lisitsyn et al., 1984; Jin and Gross, 1988; Severinov et al., 1993, 1994; Campbell et al., 2001; Garibyan et al., 2003), streptolydigin (Stl; Lisitsyn et al., 1985; Heisler et al., 1993; Severinov et al., 1993, 1995; Tuske et al., 2005; Temiakov et al., 2005), CBR703 (Artsimovitch et al., 2003; X Wang and RHE, unpublished), myxopyronin (Myx; Mukhopadhyay et al., 2008; Belogurov et al., 2009; Srivastava et al., 2011), and lipiarmycin (Lpm; Ebright, 2005; Tupin et al., 2010; Srivastava et al., 2011) (Figure 4A). The Sal target is located adjacent to, but does not overlap, the Rif, Stl, and CBR703 targets. The Sal target is distant from the Myx and Lpm targets.

### Sal does not exhibit cross-resistance with the RNAP inhibitors rifampin, streptolydigin, CBR703, myxopyronin, and lipiarmycin

Consistent with the absence of overlap between the Sal target and the Rif, Stl, CBR703, Myx, and Lpm targets, Sal-resistant mutants do not exhibit cross-resistance with Rif, Stl, CBR703, Myx, and Lpm (Figure 4B). Conversely, Rif-resistant, Stl-resistant, CBR703-resistant, Myx-resistant, and Lpm-resistant mutants do not exhibit cross-resistance with Sal (Figure 4C).

For approximately one-quarter to one-half of Sal-resistant substitutions, not only is there no cross-resistance to Stl and CBR703, but also there is significant (≥ fourfold) hyper-susceptibility to Stl and CBR703 (data in blue in Figure 4B). Resistance to a first inhibitor of an enzyme and hyper-susceptibility to a second inhibitor of the enzyme generally is understood to indicate that the two inhibitors affect different reaction steps of the enzyme and/or bind to and stabilize different conformational states of the enzyme (Tachedjian et al., 1996; Selmi et al., 2003). We infer that Sal may inhibit a different RNAP reaction step than Stl and CBR703 and/or may bind to and stabilize a different RNAP conformational state than Stl and CBR703.

### Co-administration of Sal with rifampin or myxopyronin suppresses the emergence of resistance

The absence of overlap between the Sal target and other RNAP inhibitor targets, and the absence of cross-resistance between Sal and other RNAP inhibitors, suggests that the co-administration of Sal and another RNAP inhibitor may result in an extremely low, effectively undetectable, spontaneous resistance rate, representing the product of the spontaneous resistance rate for Sal and the spontaneous resistance rate for the other RNAP inhibitor. (For many pairs of antibacterial agents having different targets and no cross-resistance, co-administration potentially results in a spontaneous resistance rate comparable to the product of the individual spontaneous resistance rates [Fischbach, 2011]. This is true even for pairs of antibacterial agents that function through the same pathway and same target protein [Fischbach, 2011].) The results in Figure 4D support this hypothesis. Thus, co-administration of Sal (resistance rate = 2 × 10^−9^ per generation) and Rif (resistance rate = 1 × 10^−9^ per generation) results in a resistance rate below the limit of detection (<2 × 10^−12^ per generation). In the same manner, co-administration of Sal (resistance rate 2 × 10^−9^ per generation) and Myx (resistance rate 3 × 10^−10^ per generation) results in a resistance rate below the limit of detection (<2 × 10^−12^ per generation). The observed suppression of the emergence of spontaneous resistance has practical implications, in view of the fact that susceptibility to spontaneous resistance is the main limiting factor in clinical use of Rif (Floss and Yu, 2005) and has been cited as a potential barrier to clinical use of Myx (Moy et al., 2011).

### Sal does not inhibit formation of a transcription initiation complex

**Figure 5.**
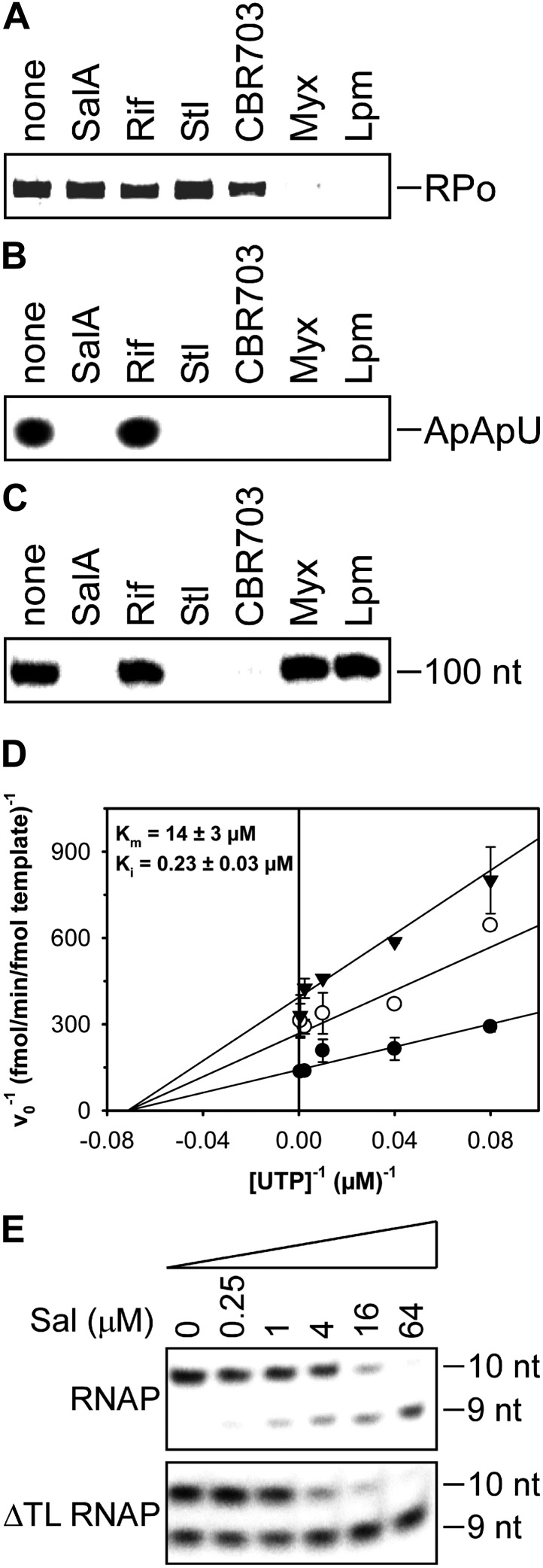
Mechanistic basis of transcription inhibition by Sal. (**A**) Sal does not inhibit formation of a transcription initiation complex (RPo). (**B**) Sal inhibits nucleotide addition in transcription initiation. (**C**) Sal inhibits nucleotide addition in transcription elongation. (**D**) Sal inhibits nucleotide addition noncompetitively. (**E**) Transcription inhibition by Sal does not require the RNAP trigger loop. **DOI:** http://dx.doi.org/10.7554/eLife.02451.007

To define the mechanistic basis of transcription inhibition by Sal, we assessed the effects of Sal on individual reaction steps in transcription initiation and transcription elongation (Figure 5).

**Figure 5—figure supplement 1.**
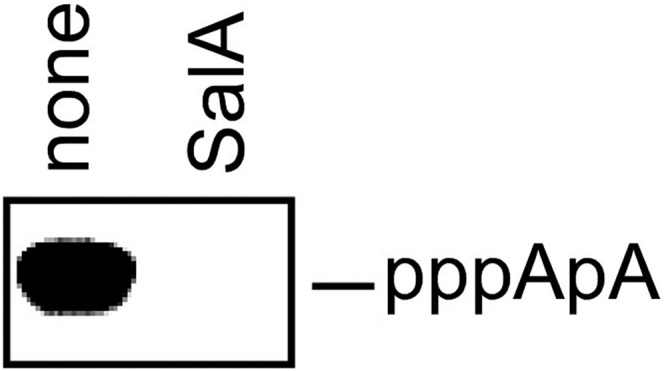
Sal inhibits nucleotide addition in de novo initiation. **DOI:** http://dx.doi.org/10.7554/eLife.02451.008

**Figure 5—figure supplement 2.**
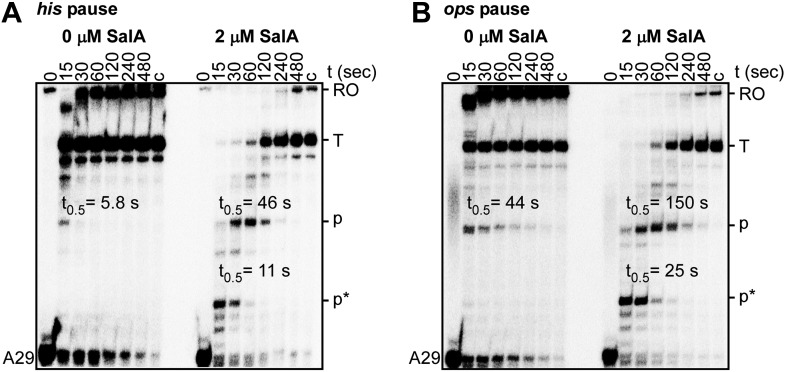
Sal enhances Type-I and Type-II transcriptional pausing. (**A**) Effects of Sal on Type-I pausing at the his pause site. (**B**) Effects of Sal on Type-II pausing at the ops pause site. A29, 29 nt RNA product in halted elongation complex; c, ‘chase’ reaction; RO, run-off RNA product; T, terminated RNA product; p, ops or his pause-site RNA product; p*, additional pause-site RNA product; t_0.5_, pause half-life. **DOI:** http://dx.doi.org/10.7554/eLife.02451.009

**Figure 5—figure supplement 3.**
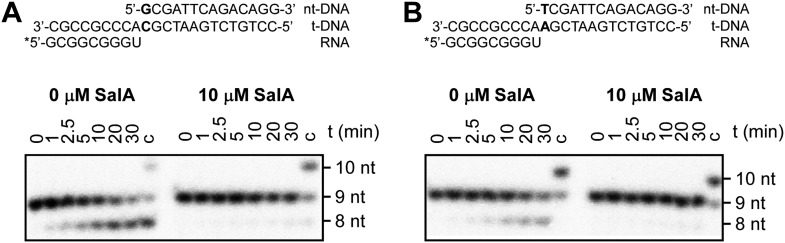
Sal inhibits pyrophosphorolysis. (**A**) Pyrophosphorolysis assay using nucleic-acid scaffold containing G:C as first base pair of downstream duplex (relatively high pyrophosphorolysis rate in absence of inhibitor). (**B**) Pyrophosphorolysis assay using nucleic-acid scaffold containing T:A as first base pair of downstream duplex (relatively low pyrophosphorolysis rate in absence of inhibitor). Gel images show pyrophosphorolysis from 0 to 30 min. 9 nt, nucleic-acid scaffold; 8 nt, product of pyrophosphorolysis; 10 nt, product of ‘chase’ reaction with GTP (left) or UTP (right); nt-DNA, DNA nontemplate strand; t-DNA, DNA template strand. **DOI:** http://dx.doi.org/10.7554/eLife.02451.010

The results in Figure 5A show that Sal does not inhibit formation of a heparin-resistant RNAP-promoter open complex. The results indicate that the mechanism of transcription inhibition by Sal differs from the mechanisms of transcription inhibition by Myx and Lpm, both of which inhibit the formation of the RNAP-promoter open complex (Mukhopadhyay et al., 2008; Belogurov et al., 2009; Tupin et al., 2010; Srivastava et al., 2011).

### Sal inhibits nucleotide addition in transcription initiation

The results in Figure 5B show that Sal inhibits nucleotide addition in transcription initiation. Sal inhibits nucleotide addition in both primer-dependent transcription initiation (Figure 5B) and de novo transcription initiation (Figure 5—figure supplement 1). In primer-dependent transcription initiation, Sal inhibits all nucleotide-addition steps, including the first nucleotide-addition step to form a 3-nt RNA product from a 2-nt RNA primer and an NTP (Figure 5B). In de novo transcription initiation, Sal inhibits all nucleotide-addition steps, including the first nucleotide-addition step to form a 2-nt RNA product from NTPs (Figure 5—figure supplement 1). The results indicate that the mechanism of transcription inhibition by Sal differs from the mechanism of transcription inhibition by Rif, which does not inhibit the first nucleotide-addition step in transcription initiation (Figure 5B; McClure and Cech, 1978).

### Sal inhibits nucleotide addition in transcription elongation

The results in Figure 5C show that Sal also inhibits nucleotide addition in transcription elongation. In transcription elongation, Sal inhibits nucleotide addition both at non-pause sites (Figure 5C) and at type-I and type-II pause sites (hairpin-stabilized pauses and backtracking-stabilized pauses; Figure 5—figure supplement 2) and inhibits not only the forward reaction of nucleotide addition but also the reverse reaction, pyrophosphorolysis (Figure 5—figure supplement 3). The results confirm that the mechanism of transcription inhibition by Sal differs from the mechanisms of transcription inhibition by Rif, Myx, and Lpm, which do not inhibit transcription elongation (Figure 5C; McClure and Cech, 1978; Mukhopadhyay et al., 2008; Belogurov et al., 2009; Tupin et al., 2010; Srivastava et al., 2011).

### Sal inhibits nucleotide addition noncompetitively

The results in Figure 5D show that inhibition by Sal is noncompetitive with respect to NTP substrate. The K_i_ for inhibition is 0.2 µM, which is equal to the IC50 for inhibition of transcription (compare Figures 5C and 1C). The results indicate that Sal does not inhibit the NTP binding sub-reaction of the nucleotide-addition cycle, but instead inhibits one or more of the bond-formation, pyrophosphate-release, and translocation sub-reactions of the nucleotide-addition cycle.

### Transcription inhibition by Sal does not require the RNAP trigger loop

The results in Figure 5E show that transcription inhibition by Sal does not require the RNAP active-center subregion referred to as the ‘trigger loop’. Thus, Sal inhibits wild-type RNAP and an RNAP-derivative having a deletion of the trigger loop to the same extent and with nearly the same concentration-dependence. These results indicate that the mechanism of transcription inhibition by Sal differs from the mechanism of transcription inhibition by Stl, which absolutely requires the RNAP trigger loop (Temiakov et al., 2005).

Taken together, the results in Figure 5 establish that Sal inhibits RNAP through a mechanism different from the mechanisms of the previously characterized RNAP inhibitors Rif, Stl, Myx, and Lpm. The observation that Sal-resistant mutants are hyper-susceptible to CBR703 (Figure 4B) suggests, but does not prove, that Sal inhibits RNAP through a mechanism that is also different from the mechanism of the previously characterized RNAP inhibitor CBR703. Based on the data presented to this point, we suggest that Sal inhibits RNAP through a novel binding site and a novel mechanism. Specifically, we suggest that Sal interacts with a binding site in the bridge-helix cap and allosterically interferes with the conformational dynamics of the BH-H_N_ required for one or more of bond formation, pyrophosphate release, and translocation in the nucleotide-addition cycle of transcription initiation and transcription elongation.

### Structural basis of transcription inhibition by Sal: crystal structures of *E. coli* RNAP holoenzyme and *E. coli* RNAP holoenzyme in complex with Sal

**Figure 6.**
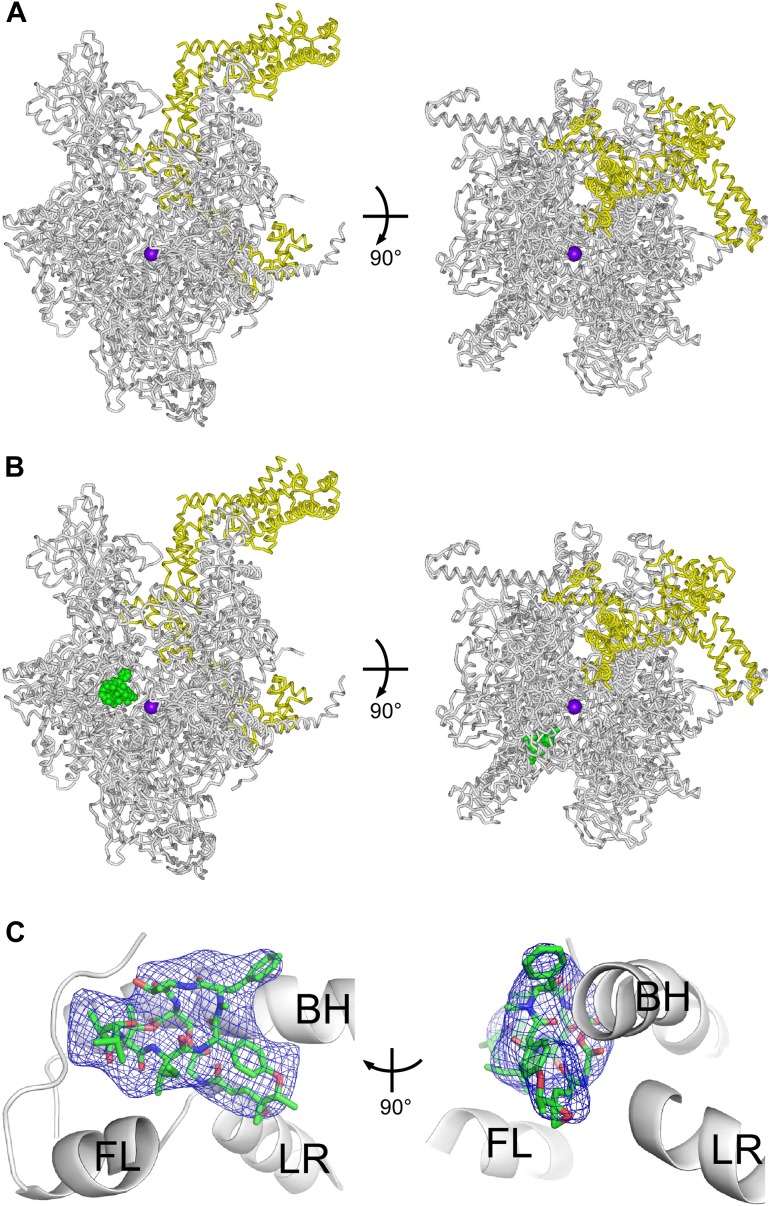
Structural basis of transcription inhibition by Sal: crystal structures of *E. coli* RNAP holoenzyme and *E. coli* RNAP holoenzyme in complex with Sal. (**A**) Structure of *E. coli* RNAP holoenzyme (two orthogonal views). Gray ribbon, RNAP core. Yellow ribbon, σ^70^. Violet sphere, active-center Mg^2+^. (**B**) Structure of *E. coli* RNAP holoenzyme in complex with Sal (two orthogonal views). Green, Sal. Other colors as in **A**. (**C**) Electron density and atomic model for Sal (two orthogonal views). Blue mesh, NCS-averaged F_o_-F_c_ omit map for Sal (contoured at 3.2σ). Green, red, and blue, Sal carbon, oxygen, and nitrogen atoms. Gray ribbons, RNAP. BH, FL, and LR, bridge helix, fork loop, and link region. **DOI:** http://dx.doi.org/10.7554/eLife.02451.011

To define the structural basis of transcription inhibition by Sal, we determined crystal structures of *E. coli* RNAP holoenzyme and *E. coli* RNAP holoenzyme in complex with Sal (Figure 6; Figure 6—figure supplement 1; Supplementary file 2). [At the time this work was performed, all published crystal structures of bacterial RNAP and bacterial RNAP complexes had employed RNAP from the genus *Thermus*. However, it was found that Sal did not inhibit RNAP from the genus *Thermus* (Figure 1C). Therefore, it was necessary to determine both a reference crystal structure of a Sal-susceptible bacterial RNAP and a crystal structure of the Sal-susceptible RNAP in complex with Sal.]

**Figure 6—figure supplement 1.**
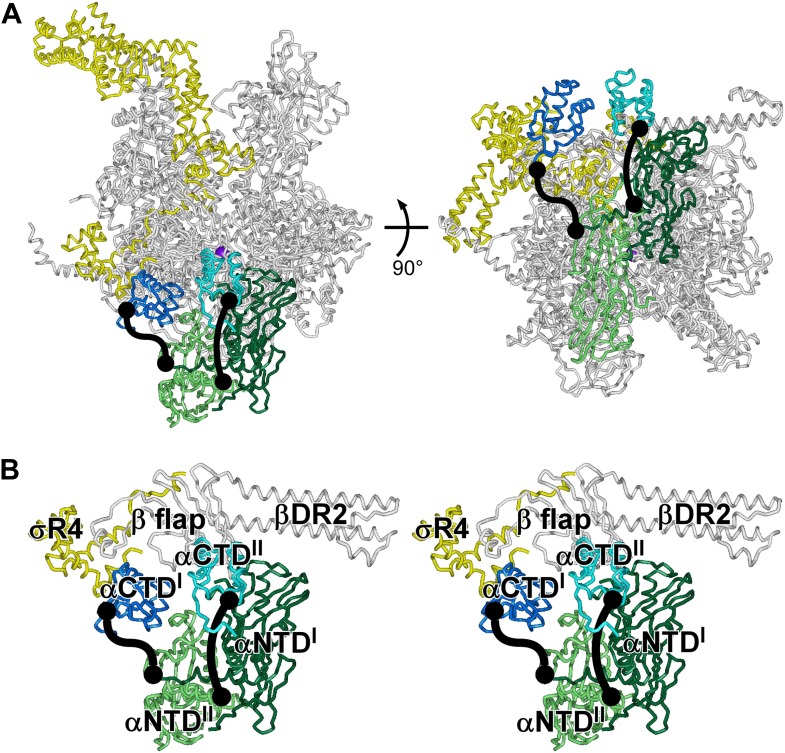
Structures of *E. coli* RNAP holoenzyme: αCTD^I^ and αCTD^II^. (**A**) Structure of *E. coli* RNAP holoenzyme (two orthogonal views). Gray, β', β and ω. Dark green and dark blue, α^I^ subunit N-terminal and C-terminal domains (αNTD^I^ and αCTD^II^). Light green and light blue, α^II^ subunit N-terminal and C-terminal domains (αNTD^II^ and αCTD^II^). Yellow, σ^70^. Violet sphere, active-center catalytic Mg^2+^. (**B**) Closeup view of αCTD^I^ and αCTD^II^ (stereoview). Gray, β flap and β dispensable region 2 (βDR2). Yellow, σ^70^ region 4 (σR4). Other colors as in **A**. **DOI:** http://dx.doi.org/10.7554/eLife.02451.012

Figure 6A shows the resulting crystal structure of *E. coli* RNAP holoenzyme at 3.9 Å resolution. In the structure, the conformations and interactions of RNAP β′ subunit, β subunit, α^I^ subunit N-terminal domain (αNTD^I^), α^II^ subunit N-terminal domain (αNTD^II^), ω subunit, and σ^70^ regions 1.2–4 in our structure match those in recently published structures of *E. coli* RNAP holoenzyme (Murakami, 2013; Zuo et al., 2013; Bae et al., 2013). Our structure also includes the α^I^ subunit C-terminal domain (αCTD^I^), with a conformation and interactions matching those in the structure of Murakami, 2013 (Figure 6—figure supplement 1). (αCTD^I^ was not present in the RNAP derivatives used for crystallization in the structures of Zuo et al., 2013 and Bae et al., 2013.) The structure also includes the α^II^ subunit C-terminal domain (αCTD^II^), positioned adjacent to, and in contact with, αNTD^I^, the β flap, and β dispensable region 2 (βDR2) (Figure 6—figure supplement 1). (αCTD^II^ was not ordered in the structure of Murakami, 2013, and was not present in the RNAP derivatives used for crystallization in Zuo et al., 2013 and Bae et al., 2013.)

Figures 6B and C show the corresponding structure of *E. coli* RNAP holoenzyme in complex with Sal at 3.9 Å resolution. The structure shows unambiguous experimental electron density for Sal in the genetically-defined Sal target, confirming the hypothesis that the genetically-defined Sal target represents the binding site for Sal on RNAP (Figure 6B,C).

### Structural basis of transcription inhibition by Sal: crystal structure of *E. coli* RNAP holoenzyme in complex with a bromine-containing Sal derivative

**Figure 7.**
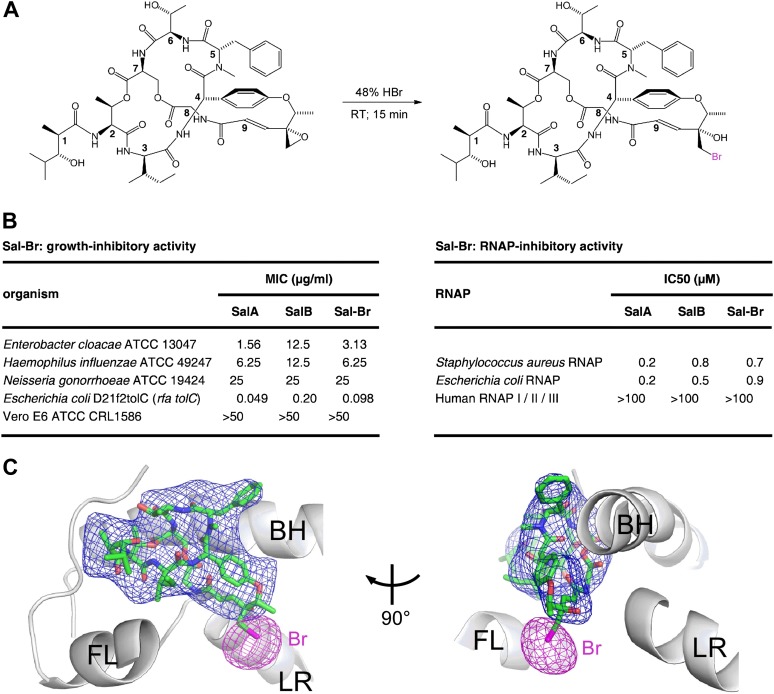
Structural basis of transcription inhibition by Sal: crystal structure of *E. coli* RNAP holoenzyme in complex with a bromine-containing Sal derivative. (**A**) Synthesis of Sal–Br. (**B**) Growth-inhibitory activity and RNAP-inhibitory activity of Sal–Br. (**C**) Electron density, Br anomalous difference density, and atomic model for Sal–Br. Blue mesh, NCS-averaged F_o_-F_c_ omit map for Sal (contoured at 3.2σ). Pink mesh, Br anomalous difference density for Sal–Br (contoured at 7.0σ). Other colors and labels as in Figure 6C. **DOI:** http://dx.doi.org/10.7554/eLife.02451.013

To confirm the binding position and binding orientation of Sal shown in Figure 6B,C, we prepared a bromine-containing Sal derivative, and collected X-ray diffraction data for *E. coli* RNAP holoenzyme in complex with the bromine-containing Sal derivative (Figure 7; Supplementary file 3). The bromine-containing Sal-derivative (‘Sal–Br’) contained a residue-9 bromohydrin moiety structurally related to the residue-9 chlorohydrin moiety of SalB (compare Figures 7A and 1A). Sal–Br was prepared by semi-synthesis from SalA, exploiting the unique chemical reactivity of the residue-9 epoxide moiety of SalA (Figure 7A). Sal–Br was found to exhibit essentially full RNAP-inhibitory activity and essentially full antibacterial activity (Figure 7B).

The RNAP–Sal–Br complex exhibited electron density for Sal–Br matching the electron density in the RNAP–Sal complex for Sal (blue mesh in Figures 6C and 7C) and exhibited a single peak of Br anomalous difference density immediately adjacent to the electron density for Sal–Br, in the position expected for a Br atom covalently bonded to a carbon atom of the Sal–Br residue-9 bromohydrin (pink mesh in Figure 7C). The results unequivocally confirm the ligand binding position and ligand binding orientation.

### Structural basis of transcription inhibition by Sal: Sal makes direct interactions with the RNAP bridge-helix cap

**Figure 8.**
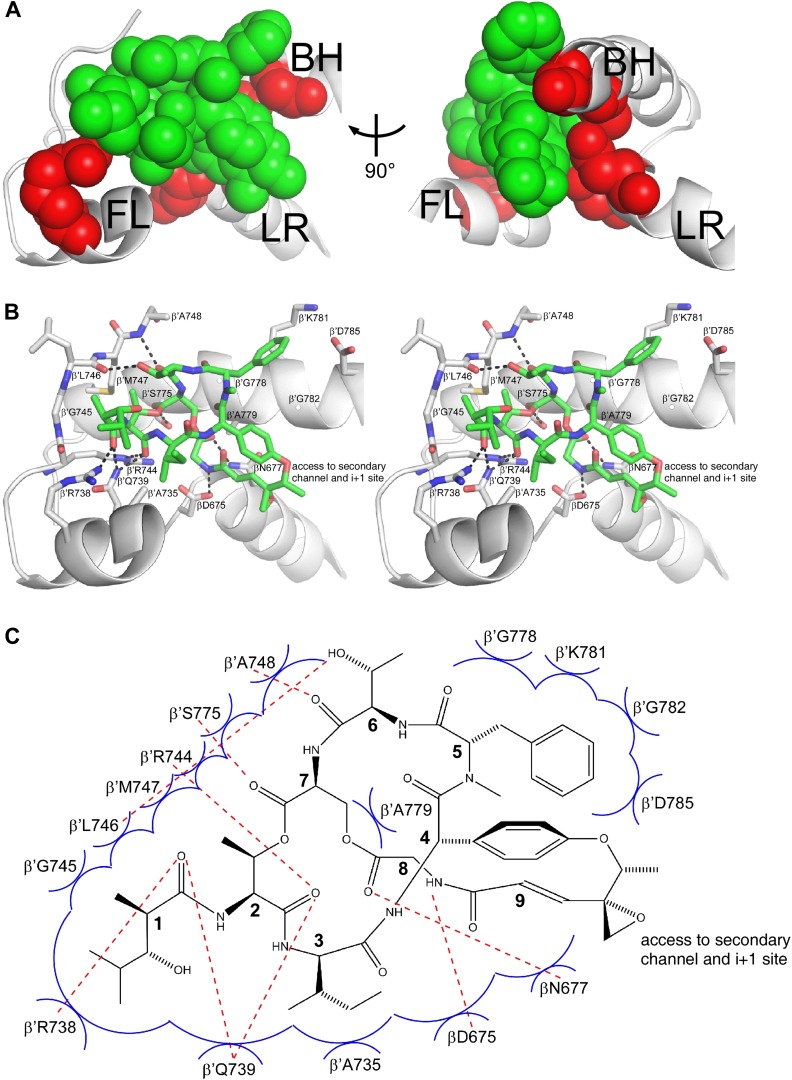
Structural basis of transcription inhibition by Sal: Sal makes direct interactions with the RNAP bridge-helix cap. (**A**) Relationship between Sal (green) and sites of substitutions that confer high-level Sal-resistance (red). Views and labels as in Figures 6C and 7C. (**B**) Contacts between RNAP and Sal (stereoview). Gray, RNAP backbone (ribbon representation) and RNAP sidechain carbon atoms (stick representation). Green, Sal carbon atoms. Red, oxygen atoms. Blue, nitrogen atoms. Dashed lines, H-bonds. (**C**) Schematic summary of inferred contacts between RNAP and Sal. Red dashed lines, H-bonds. Blue arcs, van der Waals interactions. **DOI:** http://dx.doi.org/10.7554/eLife.02451.014

The structural information shows that Sal binds within the RNAP bridge-helix cap, making direct interactions with the BH-H_N_, the fork loop, and the link region (Figures 6C, 7C, and 8). Sal makes direct interactions with all five residues at which substitutions conferring high-level (≥128-fold) Sal-resistance are obtained (β′ residues Arg738, Ala779, and Gly782, and β residues Asp675 and Asn677; red in Figure 8A). Substitution of β′ residue Arg738 would be expected to disrupt an H-bond between RNAP and Sal (Figure 8B,C). Substitution of β′ residue Ala779 or Gly782 by any residue having a larger sidechain would be expected to introduce severe steric clash between RNAP and Sal (Figure 8B,C). Substitution of β residues Asp675 and Asn677 would be expected to disrupt both H-bonds and van der Waals interactions between RNAP and Sal (Figure 8B,C). (Based on the resolution of the structure and the quality of electron density maps for residues of Sal and residues of RNAP close to Sal, the inferred proximities of individual residues of Sal to individual residues of RNAP are secure, but the inferred details of H-bonds and van der Waals interactions are, at least in part, provisional.)

**Figure 8—figure supplement 1.**
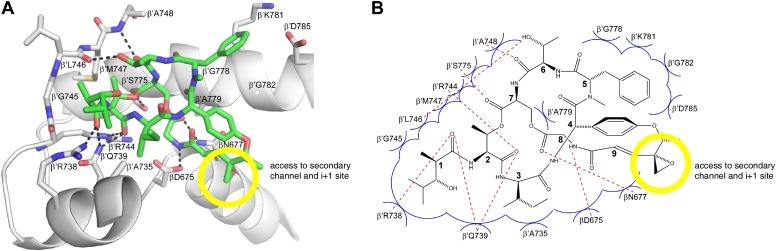
The structurally and chemically accessible epoxide moiety of SalA enables semi-synthesis of novel Sal analogs. Yellow circle, Sal residue-9 epoxide moiety. Other colors as in Figure 8B,C. **DOI:** http://dx.doi.org/10.7554/eLife.02451.015

Six of the RNAP residues that make direct contact with Sal are conserved across Gram-positive bacterial RNAP, Gram-negative bacterial RNAP, and human RNAP I, II, and III (β′ residues 739, 745, 778, 779, 782, and 785; Figures 3B and 8B,C). Nine RNAP residues that contact Sal are conserved in Gram-positive bacterial RNAP and Gram-negative bacterial RNAP, but are not conserved, and indeed are radically different, in human RNAP I, II, and III (β′ residues 738, 744, 746, 747, 748, 775, and 781, and β residues 675 and 677; Figures 3B and 8B,C). The observed interactions account for, and explain, the observation that Sal inhibits Gram-positive and Gram-negative bacterial RNAP, but does not inhibit human RNAP I, II, and III (Figure 1C).

Four of the five Sal-contacting residues in the RNAP BH-H_N_ are conserved from bacterial RNAP to human RNAP (β′ residues 778, 779, 782, and 785), presumably reflecting constraints on sequence variation imposed by the functionally essential, conformationally dynamic, BH-H_N_. In contrast, only two of the nine Sal-contacting residues in the RNAP fork loop are conserved from bacterial RNAP to human RNAP (β′ residues 739 and 745), and no Sal-contacting residues in the RNAP link region are conserved from bacterial RNAP to human RNAP, presumably reflecting lower constraints on sequence variation in these RNAP regions. The pattern of residue conservation observed for Sal is reminiscent of the pattern of residue conservation observed for the RNAP inhibitor Myx (Mukhopadhyay et al., 2008). In each case, inhibitor-contacting residues within a functionally essential, conformationally dynamic, secondary-structure element—BH-H_N_ for Sal and ‘switch 2’ for Myx—are conserved from bacterial RNAP to human RNAP, but inhibitor-contacting residues in adjacent secondary-structure elements are not, allowing for selective inhibition of bacterial RNAP but not human RNAP.

Sal binds within a ∼2000 Å^3^ pocket formed by the RNAP BH-H_N_, the RNAP fork loop, and the RNAP link region (Figure 8B,C). Backbone atoms of residues that form the pocket have superimposible conformations in RNAP holoenzyme in the absence of Sal and in RNAP holoenzyme in complex with Sal, indicating that the pocket pre-exists in RNAP holoenzyme in the absence of Sal. The pocket opens at one end onto the RNAP secondary channel and the RNAP active-center ‘i+1’ nucleotide binding site (Figure 8B,C). It seems likely that Sal enters the pocket from the RNAP secondary channel and/or the active-center i+1 nucleotide site.

Within the binding pocket, Sal residues 4, 5, 7, and 8 interact with the RNAP BH-H_N_, Sal residues 1–3 and 6–7 interact with the RNAP fork loop, and Sal residues 8 and 9 interact with the RNAP link region (Figure 8B,C). Sal residue 9 is at the end of the pocket that opens onto the RNAP secondary channel and the active-center i+1 nucleotide binding site (Figure 8B,C). The Sal residue-9 epoxide and methyl moieties extend into this opening and make no or limited interactions with RNAP (Figure 8B,C).

The interactions observed in the structure suggest an opportunity for preparation of novel Sal analogs with improved potencies by semi-synthesis. The Sal residue-9 epoxide moiety is chemically reactive (Figure 7A), can be altered without loss of activity (Figure 7B), makes no or limited interactions with RNAP (Figure 8B,C), and is directed toward the RNAP secondary channel and active-center i+1 nucleotide binding site (Figure 8B,C). Accordingly, it should be possible to prepare novel Sal derivatives by semi-synthesis, introducing sidechains at the Sal residue-9 epoxide moiety that make additional interactions with RNAP, thereby potentially increasing RNAP-inhibitory activity and antibacterial activity (Figure 8—figure supplement 1). By way of example, sidechains that carry a negative charge would be positioned to make favorable electrostatic interactions with a cluster of positively-charged residues located in the RNAP secondary channel (the ‘basic rim’; Vassylyev et al., 2007b; Zhang and Landick, 2009). By further way of example, a sidechain carrying a nucleotide, a nucleoside, or a nucleoside analog would be positioned to make highly favorable additional interactions with the RNAP active-center i+1 nucleotide binding site, potentially enabling highly potent RNAP-inhibitory activity and antibacterial activity.

### Structural basis of transcription inhibition by Sal: Sal interacts with an ‘open’ (unbent) state of the bridge-helix N-terminal hinge and an ‘open’ (unfolded) state of the trigger loop

**Figure 9.**
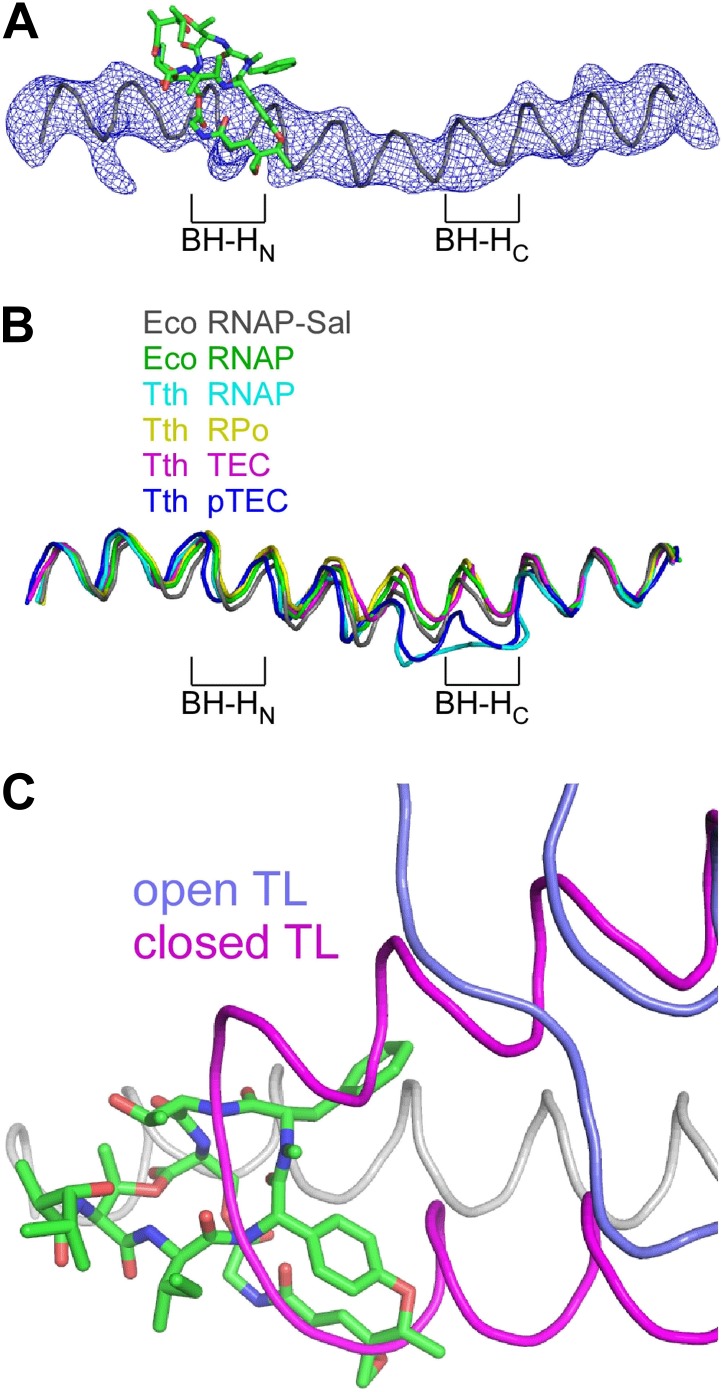
Structural basis of transcription inhibition by Sal: Sal interacts with an ‘open’ (unbent) state of the bridge-helix N-terminal hinge and an ‘open’ (unfolded) state of the trigger loop. (**A**) Electron density and model for bridge helix in crystal structure of RNAP-Sal. Blue mesh, F_o_-F_c_ omit map for bridge helix (contoured at 2.5σ). Black ribbon, bridge-helix backbone. Green, red, and blue, Sal carbon, oxygen, and nitrogen atoms. BH-H_N_, bridge-helix N-terminal hinge. BH-H_C_, bridge-helix C-terminal hinge. (**B**) Superimposition of bridge helices of *E. coli* RNAP-Sal (black), *E. coli* RNAP (green; unbent BH-H_N_ and BH-H_C_), *T. thermophilus* RNAP (cyan; PDB 1IW7), *T. thermophilus* RPo (yellow; PDB 4G7H), *T. thermophilus* transcription elongation complex (pink; PDB 2O5J), and paused *T. thermophilus* transcription elongation complex (violet; PDB 4GZY). (**C**) Predicted absence of steric clash between Sal (colors as in **A**) and trigger loop in open conformational state (blue; PDB 1ZYR) and predicted presence of steric clash between Sal and trigger loop in closed conformational state (pink; PDB 2O5J). **DOI:** http://dx.doi.org/10.7554/eLife.02451.016

The crystal structure of the RNAP-Sal complex also defines effects of Sal on RNAP conformation (Figure 9). The crystal structure shows that Sal interacts with the RNAP BH-H_N_ in an open (unbent) state (Figure 9A), the same state that has been observed in previous crystal structures of RNAP and RNAP complexes (Zhang et al., 1999, 2012; Campbell et al., 2001; Vassylyev et al., 2002, 2007a, 2007b; Temiakov et al., 2005; Tuske et al., 2005; Mukhopadhyay et al., 2008; Belogurov et al., 2009; Murakami, 2013; Zuo et al., 2013; Bae et al., 2013; Figure 9B). This conformation is different from the closed (bent) BH-H_N_ conformation that has been observed in molecular dynamics simulations of nucleotide-addition reactions in transcription elongation complexes (Weinzierl, 2010; Kireeva et al., 2012; Nedialkov et al., 2013), and that has been postulated to serve as a critical intermediate in the bond-formation, pyrophosphate-release, and/or translocation reactions of the nucleotide-addition cycle (Hein and Landick, 2010; Weinzierl, 2010; Kireeva et al., 2012; Nedialkov et al., 2013). We conclude that Sal interacts with an open (unbent) BH-H_N_ conformational state, and we propose that, through its interactions with that state, it stabilizes that state and prevents conformational dynamics required for nucleotide addition.

In the crystal structure of the RNAP-Sal complex, the RNAP trigger loop is disordered. Molecular modelling indicates that the structure of RNAP-Sal is compatible with the open (unfolded) trigger-loop conformations observed in crystal structures of RNAP and the transcription elongation complex without a bound NTP substrate (Zhang et al., 1999; Campbell et al., 2001; Vassylyev et al., 2002, 2007a; Temiakov et al., 2005; Tuske et al., 2005; Mukhopadhyay et al., 2008; Belogurov et al., 2009; Murakami, 2013; Zuo et al., 2013; Bae et al., 2013), but would be incompatible with the closed (folded) trigger loop conformation observed in the crystal structure of the transcription elongation complex with a bound NTP substrate (Vassylyev et al., 2007b; Figure 9C). We infer that Sal interacts with an open (unfolded) trigger-loop conformational state, and likely would prevent the formation of the closed (folded) trigger-loop conformational state. It is possible that effects of Sal on trigger-loop conformation may contribute to the mechanism of transcription inhibition by Sal. However, the results in Figure 5E show that the trigger loop is not essential for transcription inhibition by Sal, and therefore, although effects of Sal on trigger loop conformation may contribute to transcription inhibition by Sal, they cannot be essential for transcription inhibition by Sal.

## Discussion

### Bacterial RNAP is the functional cellular target of Sal

The results in Figure 2 show that Sal inhibits RNAP in bacterial cells in culture, and that Sal-resistant mutations occur in RNAP subunit genes. The results establish that the RNAP is the functional cellular target of Sal, confirming the hypothesis that the RNAP-inhibitory activity of Sal is responsible for the antibacterial activity of Sal.

### Sal interacts with the RNAP bridge-helix cap

The results in Figure 3 establish that transcription inhibition by Sal requires a determinant located within the RNAP active-center bridge-helix cap and comprising residues of the RNAP BH-H_N_, the RNAP F-loop, and the RNAP link region (‘Sal target’). The results in Figure 4 establish that the Sal target is different from, and does not overlap, the targets of the previously characterized RNAP inhibitors Rif, Stl, CBR703, Myx, and Lpm. Consistent with the absence of overlap, mutants resistant to Sal are not cross-resistant with these other RNAP inhibitors, and, reciprocally, mutants resistant to these other RNAP inhibitors are not cross-resistant with Sal. Consistent with the absence of cross-resistance, co-administration of Sal and Rif, or of Sal and Myx, suppresses the emergence of spontaneous resistance, a finding that is significant since emergence of resistance limits the clinical application of Rif (Floss and Yu, 2005) and has been cited as a potential obstacle to the clinical development of Myx (Moy et al., 2011).

### Sal inhibits nucleotide addition in transcription initiation and transcription elongation

The results in Figure 5 establish that Sal inhibits nucleotide addition in both transcription initiation and transcription elongation, interfering with one or more of the bond-formation, pyrophosphate-release, or translocation sub-reactions of the nucleotide-addition cycle. The results in Figure 5 show that the mechanism of inhibition by Sal is different from the mechanisms of inhibition by the previously characterized RNAP inhibitors Rif, Stl, Myx, and Lpm; and further results in Figure 4B suggest, although do not prove, that the mechanism of Sal also is different from the mechanism of the previously characterized RNAP inhibitor CBR703.

### Sal allosterically inhibits nucleotide addition through interaction with the bridge-helix cap trapping an ‘open’ (unbent) state of the bridge-helix N-terminal hinge

The crystal structures of RNAP–Sal and RNAP–Sal–Br complexes in Figures 6–8 confirm that Sal binds within the RNAP bridge–helix cap, making interactions with residues of the BH-H_N_, the F-loop, and the link region. The structures establish that Sal does not contact, or clash with, the RNAP active-center catalytic Mg^2+^ ion or the RNAP residues that interact with the DNA template, the RNA product, or the NTP substrate, indicating that Sal interferes with nucleotide addition allosterically. The structures further reveal that Sal interacts with an open (unbent) state of the BH-H_N_ (Figure 8A). We propose that Sal allosterically inhibits nucleotide addition by interacting with and stabilizing the open (unbent) state of the BH-H_N_.

### Sal as a chemical probe of bridge-helix N-terminal hinge conformation

Sal is the first RNAP inhibitor that has been proposed to function through effects on conformational dynamics of the BH-H_N_. We suggest that Sal will find use as a research tool for dissection of mechanistic and structural aspects of BH-H_N_ conformational dynamics.

### Sal as a starting point for antibacterial drug discovery

The semi-synthesis of Sal–Br from SalA (Figure 7A) shows that the SalA epoxide moiety provides a chemical reactivity that can be exploited for semi-synthesis of novel Sal analogs. The retention of RNAP inhibitory activity and antibacterial activity by Sal–Br (Figure 7B) shows that semi-synthetic modifications at the SalA epoxide moiety can be tolerated without loss of potency. The crystal structures of RNAP–Sal and RNAP–Sal–Br (Figures 6–8) show that the SalA epoxide moiety makes no or limited interactions with RNAP and is located at the entrance to the Sal-binding pocket, directed towards the RNAP secondary channel and RNAP active-center i+1 nucleotide binding site (Figure 8B,C; Figure 8—figure supplement 1). These findings, together with the published total synthesis of SalA (Tan and Ma, 2008), set the stage for rational, structure-based design of novel semi-synthetic and fully synthetic Sal analogs with increased potency. Introduction at the SalA epoxide moiety of a sidechain with negatively-charged functionality should enable new, energetically favorable, electrostatic interactions with positively-charged ‘basic-rim’ residues in the RNAP secondary channel. Introduction at the Sal epoxide moiety of a nucleotide or nucleoside analog, should enable new, energetically favorable, interactions with the RNAP active-center i+1 nucleotide binding site. Covalently linking Sal to a nucleotide or nucleoside analog is expected to yield a bipartite inhibitor that interacts simultaneously with the Sal binding pocket and the active-center i+1 nucleotide binding site, and therefore, that potentially exhibits a very high affinity of binding and a very high potency of inhibition. Reciprocally, equipping a nucleoside-analog RNAP inhibitor with chemical functionality able to interact with the Sal pocket should provide a means both to increase potency of the nucleoside-analog inhibitor and to introduce selectivity for inhibition of bacterial RNAP vs inhibition of human RNAP.

## Materials and methods

### Sal

SalA and SalB were prepared from cultures of *Streptomyces* sp. CNB-091 as in Moore et al. (1999).

### Sal–Br

Sal–Br was prepared by adding 48% HBr (10 μl; 89 μmol; Sigma–Aldrich, St. Louis, MO) to SalA (5 mg; 4.9 μmol) in 250 μl chloroform and stirring 15 min at 24°C. The reaction mixture was washed with 100 μl saturated sodium bicarbonate, and the organic layer was separated, washed with 100 ml water, dried with anhydrous Na_2_SO_4_, and evaporated to a white solid. The resulting solid was purified via silica flash chromatography (0–10% methanol in chloroform). Yield: 5 mg, 93%. MS (MALDI): calculated: *m/z* 1122.456, 1124.454 (M+Na^+^); found: 1122.481, 1123.486, 1124.480, 1125.492.

### *E. coli* RNAP holoenzyme

*E. coli* strain XE54 (Tang et al., 1994) was transformed with plasmid pREII-NHα (encodes N-terminally-hexahistidine-tagged *E. coli* RNAP α subunit under control of tandem *lpp* and *'lacUV5* promoters; Niu et al., 1996). A a single colony of the resulting transformant strain was used to inoculate 50 ml fermentation broth (FB; 32.5 mM Na_2_HPO_4_, 17.4 mM KH_2_PO_4_, 5 mM MgSO_4_, 12 g/l tryptone, 24 g/l yeast extract, and 5 g/l glucose; pH 7.1) containing 200 mg/l ampicillin in a 200 ml flask, and the culture was incubated 16 hr at 37°C with shaking. A 50 ml aliquot of the culture was used to inoculate 2.8 L FB containing 200 mg/l ampicillin and 0.5 ml of polypropylene glycol 2000 as antifoam in a Minifors 5 L bioreactor (INFORS HT, Bottmingen, Switzerland), and fermentation was carried out at 37°C with stirring (800 rpm) and with maintenance of O_2_ (fresh air inlet and exhaust air outlet; air flow rate governed by O_2_ sensor), pH (10 M NaOH supply; flow rate governed by pH sensor), and nutrient (50% glycerol supply; flow rate equal to flow rate of 10 M NaOH) (procedures essentially as in Riek et al., 2008). When the culture reached OD_600_ = 10, the culture was induced by addition of IPTG to 1 mM, and fermentation was continued for 3 hr at 37°C. Cells were harvested by centrifugation (5000×*g*; 30 min at 4°C) and stored at −80°C. Cells were lysed, and RNAP holoenzyme was purified using procedures in Niu et al. (1996). Following the ammonium-sulfate-precipitation step, the pellet was dissolved in 100 ml buffer A (10 mM Tris–HCl, pH 7.9, 500 mM NaCl, 10 mM β-mercaptoethanol, and 5% glycerol), loaded onto 8 ml Ni-NTA Agarose (Qiagen, Venlo, Netherlands), washed with 50 ml buffer A containing 5 mM imidazole, washed with 50 ml buffer A containing 10 mM imidazole, and eluted with 50 ml buffer A containing 150 mM imidazole. The eluate was diluted with equal volume of buffer B (10 mM Tris–HCl, pH 7.9, 1 mM EDTA, 1 mM DTT, and 5% glycerol) and purified by anion-exchange chromatography on a 16/10 Mono Q column (GE Healthcare, Piscataway, NJ; 160 ml linear gradient of 300–500 mM NaCl in buffer B; flow rate = 1 ml/min). Fractions containing RNAP holoenzyme were pooled, concentrated to 2 ml using Amicon Ultra-15 centrifugal filters (Millipore, Billerica, MA), loaded onto a HiLoad 16/600 Superdex 200 column (GE Healthcare) pre-equilibrated in buffer C (10 mM Tris–HCl, pH 7.9, 100 mM NaCl, and 1% glycerol), and eluted with 120 ml of buffer C. Fractions containing RNAP holoenzyme were pooled, concentrated to 10 mg/ml using Amicon Ultra-15 centrifugal filter (Millipore) and stored at −80°C. Yields were 5 mg/l, and purities were >95%.

### *E. coli* RNAP core enzyme

RNAP core was prepared in the same manner, but using *E. coli* strain BL21(DE3) (Invitrogen, Carlsbad, CA) transformed with plasmids pEcABC-H6 (encodes RNAP α subunit, β subunit, and N-terminally hexahistidine-tagged β' subunit under control of the bacteriophage T7 gene 10 promoter; Hudson et al., 2009) and pCDFω (encodes RNAP ω subunit; under control of the bacteriophage T7 gene 10 promoter; Vrentas et al., 2005). ΔTL RNAP core was prepared in the same manner, but using BL21(DE3) transformed with plasmids pRL4455-β'Δ(931-1137)ΩAla3 (encodes RNAP α subunit, β subunit, C-terminally hexahistidine-tagged β' subunit with residues 931–1137 replaced by Ala-Ala-Ala, and ω subunit, under control of the bacteriophage T7 gene 10 promoter; Toulokhonov et al., 2007) and pCDFω. Yields were 10 mg/l, and purities were >95%.

### Growth-inhibitory activity

Minimum inhibitory concentrations (MICs) were quantified using broth microdilution assays as in Clinical and Laboratory Standards Institute (2009), using a starting cell density of 2–5 × 10^5^ cfu/ml, Mueller Hinton II cation adjusted broth (BD Biosciences, San Jose, CA), and an air atmosphere for *Enterobacter cloacae*, *Pseudomonas aeruginosa, Bacillus anthracis*, *Burkholderia mallei*, and *Yersinia pestis*, and using a starting cell density of 2–5 × 10^5^ cfu/ml, Haemophilus Test Medium broth (Barry et al., 1993) and a 7% CO_2_, 6% O_2_, 4% H_2_, 83% N_2_ atmosphere for *Haemophilus influenzae, Neisseria gonorrhoeae, and Moraxella catarrhalis*.

MICs for mammalian cells (Vero E6) in culture were quantified using CellTiter96 assay (Promega, Madison, WI; procedures as specified by the manufacturer).

### RNAP-inhibitory activity

Reaction mixtures contained (10 μl): 0-100 μM test compound, bacterial RNAP holoenzyme (75 nM *E. coli* RNAP holoenzyme, 75 nM *S. aureus* RNAP core enzyme and 300 nM *S. aureus* σ^A^ [prepared as in Srivastava et al., 2011], or 75 nM *T. thermophilus* RNAP holoenzyme [prepared as in Zhang et al., 2012], 20 nM DNA fragment N25-*lacUV5-*14 (positions −100 to −1 of the bacteriophage T5 N25 promoter [Gentz and Bujard, 1985] followed by positions +1 to +29 of the *lacUV5*(+10A;+15C) promoter; prepared by PCR amplification of a synthetic nontemplate-strand oligodeoxyribonucleotide), 0.5 mM ApA, 100 μM [α^32^P]UTP (0.2 Bq/fmol), 100 μM ATP, and 100 μM GTP in TB (50 mM Tris–HCl, pH 8.0, 100 mM KCl, 10 mM MgCl_2_, 1 mM DTT, 10 μg/ml acetylated bovine serum albumin, 5% methanol, and 5% glycerol). Reaction components except DNA, ApA, and NTPs were pre-incubated 10 min at 24°C; DNA was added and reaction mixtures were incubated 10 min at 37°C; ApA, 0.15 μl 7 μM [α^32^P]UTP (200 Bq/fmol), ATP, and GTP were added and reaction mixtures were incubated 5 min at 37°C; and 0.5 μl 2 mM UTP was added and reaction mixtures were incubated 5 min at 37°C. Reactions were terminated by adding 10 μl loading buffer (80% formamide, 10 mM EDTA, 0.02% bromophenol blue, and 0.02% xylene cyanol) and heating 2 min at 95°C. Products were applied to 7 M urea 15% polyacrylamide (19:1 acrylamide:bisacrylamide) slab gels (Bio-Rad, Hercules, CA), electrophoresed in TBE (90 mM Tris-borate, pH 8.0, and 2 mM EDTA), and analyzed by storage-phosphor scanning (Typhoon; GE Healthcare). Data shown are means of at least two determinations.

Radiochemical assays with human RNAP I/II/III were performed essentially as in Sawadogo and Roeder (1985). Reaction mixtures contained (20 µl): 0-100 µM test compound, 8 U HeLaScribe Nuclear Extract (Promega), 1 µg human placental DNA (Sigma-Aldrich), 400 μM ATP, 400 μM [α^32^P]UTP (0.11 Bq/fmol), 400 μM CTP, 400 μM GTP, 50 mM Tris–HCl, pH 8.0, 7 mM HEPES-NaOH, 70 mM (NH_4_)_2_SO_4_, 50 mM KCl, 12 mM MgCl_2_, 5 mM DTT, 0.1 mM EDTA, 0.08 mM phenylmethylsulfonyl fluoride, 5% methanol, and 16% glycerol. Reaction components other than DNA and NTPs were pre-incubated 10 min at 30°C, DNA was added and reaction mixtures were incubated 15 min at 30°C, NTPs were added and reaction mixtures were incubated 60 min at 30°C. Reaction mixtures were spotted on DE81 filter discs (Whatman, Kent, UK; pre-wetted with water) and incubated 1 min at room temperature. Filters were washed with 3 × 3 ml Na_2_HPO_4_, 2 × 3 ml water, and 3 ml ethanol, using a filter manifold (Hoefer, Holliston, MA). Filters were placed in scintillation vials containing 10 ml Scintiverse BD Cocktail (Thermo Fisher, Waltham, MA), and radioactivity was quantified by scintillation counting (LS6500; Beckman–Coulter, Brea, CA).

Half-maximal inhibitory concentrations (IC50s) were calculated by non-linear regression in SigmaPlot (SPSS, Chicago, IL).

### Macromolecular synthesis

Macromolecular synthesis assays were performed essentially as in Cotsonas King and Wu, 2009. *E. coli* D21f2tolC (Fralick and Burns-Keliher, 1994) was cultured in 10 ml LB broth (Sambrook and Russell, 2001) at 37°C with shaking until OD_600_ = 0.4–0.8, and cultures were diluted with LB broth to OD_600_ = 0.167. Aliquots (90 µl) were dispensed into wells of a 96-well plate; were supplemented with 7 µl of pre-warmed 4 µCi/ml [^14^C]-thymidine, 10 µCi/ml [^14^C]-uracil, or 30 µCi/ml [^14^C]-amino acid mix (PerkinElmer, Waltham, MA); were incubated 10 min at 37°C with shaking; were supplemented with 3 µl 3.3 µg/ml SalA in methanol (final concentration = 2 × MIC), 3 µl 13 µg/ml Rif in methanol (final concentration = 2 × MIC), or 3 µl solvent blank; and incubated at 37°C with shaking. At time points 0, 10, 20, and 30 min after the addition of SalA, Rif, or solvent blank, rows of samples were transferred to a second 96-well plate, containing 100 µl ice-cold 10% trichloroacetic acid (TCA) in each well, and the second plate was incubated on ice. 1 hr after the final time point, TCA precipitates were collected by filtration onto glass-fiber filters (Filtermat A; PerkinElmer; pre-rinsed twice with 5% TCA), washed with 2 × ∼300 µl 5% TCA, washed with 3 × ∼300 µl water, and washed with 2 × ∼300 µl 10% ethanol, using a Packard FilterMate 196 Cell Harvester with Matrix Filter upper head assembly (PerkinElmer). Filters were dried under a heat lamp, wrapped in a single layer of plastic wrap, and exposed to a storage phosphor screen for 18–19 hr, and analyzed by storage-phosphor scanning (Typhoon; GE Healthcare).

### Spontaneous Sal-resistant mutants

*E. coli* D21f2tolC was cultured to saturation in 5 ml LB broth at 37°C, cultures were centrifuged, and cell pellets (∼2 × 10^9^ cells) were re-suspended in 50 μl LB broth and plated on LB agar (Sambrook and Russell, 2001) containing 0.6 or 1.2 μg/ml SalA (2 × MIC or 4 × MIC under these conditions), and incubated 24–48 hr at 37°C. Sal-resistant mutants were identified by the ability to form colonies on this medium and were confirmed by re-streaking on the same medium.

Genomic DNA was isolated using the Wizard Genomic DNA Purification Kit (Promega; procedures as specified by the manufacturer) and was quantified by measurement of UV-absorbance (procedures as in Sambrook and Russell, 2001). The *rpoC* gene and the *rpoB* gene were PCR-amplified in reactions containing 0.2 µg genomic DNA, 0.4 µM forward and reverse oligodeoxyribonucleotide primers (5′-AGGTCACTGCTGTCGGGTTAAAACC-3′ and 5′-TGACAAATGCTCTTTCCCTAAACTCC-3′ for *rpoC*; 5′-GTTGCACAAACTGTCCGCTCAATGG-3′ and 5′-TCGGAGTTAGCACAATCCGCTGC-3′ for *rpoB*), 5 U Taq DNA polymerase (Genscript, Piscataway, NJ), and 800 µM dNTP mix (200 µM each dNTP; Agilent/Stratagene, La Jolla, CA) (initial denaturation step of 3 min at 94°C; 30 cycles of 30 s at 94°C, 45 s at 52°C, and 4.5 min at 68°C; final extension step of 10 min at 68°C). PCR products containing the *rpoC* gene (4.2 kB) or the *rpoB* gene (4.0 kB) were isolated by electrophoresis on 0.8% agarose (procedures as in Sambrook and Russell, 2001), extracted from gel slices using a Gel/PCR DNA Fragments Extraction Kit (IBI Scientific, Peosta, IA; procedures as specified by the manufacturer), and submitted to the High Throughput Genomics Center (Seattle, WA) for sequencing (Sanger sequencing; eight sequencing primers per gene).

### Induced Sal-resistant mutants

Induced Sal-resistant mutants were isolated using procedures analogous to those used for isolation of induced Myx-resistant mutants in Mukhopadhyay et al. (2008). Random mutagenesis of *rpoB* plasmid pRL706 (Severinov et al., 1997) and *rpoC* plasmid pRL663 (Wang et al., 1995) was performed by use of PCR amplification, exploiting the baseline error rate of PCR amplification. Mutagenesis reactions were performed using the QuikChange Site-Directed Mutagenesis Kit (Agilent/Stratagene), with pRL706 as template and oligodeoxyribonucleotide forward and reverse primers corresponding to nucleotides 427-446 of *lacI* (5′-GTTCCGGCGTTATTTCTTGA-3′ and 5′-TCAAGAAATAACGCCGGAAC-3′), or with pRL663 as template and oligodeoxyribonucleotide forward and reverse primers corresponding to nucleotides 217-246 of *lacI* (5′-CTGCACGCGCCGTCGAAAATTGTCGCGGCG-3′ and 5′-CGCCGCGACAATTTTCGACGGCGCGTGCAG-3′) (primers at 160 nM; all other components at concentrations as specified by the manufacturer). Mutagenized plasmid DNA was introduced by transformation into *E. coli* XL1-Blue (Agilent/Stratagene). Transformants (∼5 × 10^3^ cells) were applied to LB-agar plates containing 200 μg/ml ampicillin, plates were incubated 16 hr at 37°C, and plasmid DNA was prepared from the pooled resulting colonies. The resulting passaged random-mutagenesis library was pooled in a 1/1 (wt/wt) ratio with pooled passaged saturation-mutagenesis libraries of Mukhopadhyay et al. (2004), Tuske et al. (2005), and Mukhopadhyay et al. (2008), and the resulting pooled mutagenized plasmid DNA was introduced by transformation into *E. coli* D21f2tolC. Transformants (∼10^3^ cells) were applied to LB-agar plates containing 0.4 μg/ml SalA (for pRL706) or 1 μg/ml SalA (for pRL663), 200 μg/ml ampicillin, and 1 mM IPTG, and plates were incubated 24–48 hr at 37°C. Sal-resistant mutants were identified by the ability to form colonies on this medium, were confirmed by re-streaking on the same medium, and were demonstrated to contain plasmid-linked Sal-resistant mutations by preparing plasmid DNA, transforming *E. coli* D21f2tolC with plasmid DNA, and plating transformants on the same medium. Nucleotide sequences of *rpoB* and *rpoC* were determined by Sanger sequencing (eight primers per gene).

### Complementation assays

For complementation assays with *rpoC* derivatives, temperature-sensitive *E. coli* strain 397c (*rpoC*^*ts*^*397 argG thi lac* [λcI_857_h_80_S_t68_d*lac*^+^]; Christie et al., 1996) was transformed with pRL663 or a pRL663 derivative, transformants (10^3^–10^4^ cells) were applied to LB-agar plates containing 200 μg/ml ampicillin and 1 mM IPTG, plates were incubated 22 hr at 43°C, and bacterial growth was scored. For complementation assays with *rpoB* derivatives, temperature-sensitive *E. coli* strain RL585 (*rpoB*^*am*^*cI supD*^*ts*^*43,74* Δ*(recA-srl)306 lacZ*^*am*^*2110 galEK*^*am*^ *leu*^*am*^ *trp*^*am*^ *sueA rpsL tsx srl301*::Tn*10-84*; Landick et al., 1990) was transformed with pRL706 or a pRL706 derivative, transformants (10^3^–10^4^ cells) were applied to LB-agar plates containing 200 μg/ml ampicillin, 1 mM IPTG, and 10 μg/ml tetracycline, plates were incubated 22 hr at 43°C, and bacterial growth was scored.

### Resistance levels

Resistance levels of Sal-resistant mutants were quantified by performing broth microdilution assays. Single colonies were inoculated into 5 ml LB broth (LB broth containing 200 μg/ml ampicillin for induced mutants and wild-type controls for induced mutants) and incubated at 37°C with shaking until OD_600_ = 0.4–0.8. (At this point, IPTG was added to a final concentration of 1 mM for induced mutants and wild-type controls for induced mutants, and the cultures were grown for an additional 1 hr at 37°C with shaking.) Diluted aliquots (∼5 × 10^4^ cells in 97 μl LB broth; LB broth containing 200 μg/ml ampicillin and 1 mM IPTG for induced mutants and wild-type controls for induced mutants) were dispensed into wells of a 96-well plate, were supplemented with 3 μl of a twofold dilution series of SalA or SalB in methanol (final concentrations = 0.0015–50 μg/ml), or 3 μl of a solvent blank, and were incubated 16 hr at 37°C with shaking. The MIC was defined as the lowest tested concentration of SalA that inhibited bacterial growth by ≥90%.

### Cross-resistance levels

Cross-resistance levels of Sal-resistant mutants were determined analogously to resistance levels, using 0.003–200 μg/ml Rif (Sigma–Aldrich), Stl (Sourcon-Padena, Tübingen, Germany), CBR703 (Maybridge, Tintagel, UK), MyxB (synthesized as in Ebright and Ebright, 2013), and Lpm (BioAustralis, Smithfield, Australia).

Cross-resistance levels of Rif-resistant mutants, Myx-resistant mutants, and Lpm-resistant mutants (mutations transferred from pRL706 or pRL663 derivatives [Ebright, 2005; Mukhopadhyay et al., 2008; DD, S Ismail and RHE, unpublished] to the chromosome of *E. coli* D21f2tolC by λ-Red-mediated recombineering [procedures essentially as in Datsenko and Wanner, 2000, but using transformation rather than electroporation]) were determined analogously to resistance levels of spontaneous Sal-resistant mutants. Cross-resistance levels of Stl-resistant mutants and CBR703-resistant mutants (mutations on pRL706 and pRL663 derivatives; Tuske et al., 2005; X Wang and RHE, unpublished) were determined analogously to resistance levels of induced Sal-resistant mutants.

### Resistance rates

Resistance rates were determined using fluctuation assays essentially as in Srivastava et al. (2012). Defined numbers of cells of *E. coli* D21f2tolC (10^8^–10^11^ cfu/plate) were plated on LB agar containing 0.6 μg/ml (2 × MIC) SalA, 1 μg/ml (2 × MIC) Rif, 6 μg/ml (2 × MIC) MyxB, both 0.6 μg/ml SalA and 1 μg/ml Rif, or both 0.6 μg/ml SalA and 6 μg/ml MyxB, and numbers of colonies were counted after 24 hr at 37°C (at least five independent determinations each). Resistance rates and 95% confidence intervals were calculated using the Ma-Sandri-Sarkar Maximum Likelihood Estimator (Ma et al., 1992; Sarkar et al., 1992) as implemented on the Fluctuation Analysis Calculator (http://www.keshavsingh.org/protocols/FALCOR.html) (Hall et al., 2009).

### Formation of RNAP-promoter open complex

Reaction mixtures contained (20 μl): test compound (0 or 10 μM SalA, 0.2 μM Rif, 400 μM Stl, 100 μM CBR703, 20 μM MyxB, or 100 μM Lpm), 40 nM *E. coli* RNAP holoenzyme, 10 nM DNA fragment containing positions −42 to +426 of the *lacUV5(ICAP)* promoter (Naryshkin et al., 2001), and 100 μg/ml heparin in TB. Reaction components other than DNA and heparin were pre-incubated 5 min at 24°C, DNA was added and reaction mixtures were incubated 15 min at 37°C; heparin was added and reactions were incubated 2 min at 37°C to disrupt non-specific RNAP-promoter complexes and RNAP-promoter closed complexes (Cech and McClure, 1980). Products were applied to 5% TBE polyacrylamide slab gels (Bio-Rad), gels were electrophoresed in TBE, and gels were stained with SYBR Gold Nucleic Acid Gel Stain (Life Technologies, Grand Island, NY).

### Nucleotide addition in transcription initiation: primer-dependent initiation

Reaction mixtures contained (10 μl): test compound (0 or 10 μM SalA, 0.2 μM Rif, 400 μM Stl, 100 μM CBR703, 20 μM MyxB, or 100 μM Lpm), 40 nM *E. coli* RNAP holoenzyme, 10 nM DNA fragment containing positions −42 to +426 of the *lacUV5(ICAP)* promoter (Naryshkin et al., 2001), 0.5 mM ApA, and 10 μM [α^32^P]UTP (1.5 Bq/fmol) in TB. Reaction components except DNA, ApA, and [α-^32^P]UTP were pre-incubated 10 min at 37°C, DNA was added and reaction mixtures were incubated 10 min at 37°C, ApA and [α^32^P]UTP were added and reaction mixtures were incubated 10 min at 37°C. Reactions were terminated by adding 10 μl loading buffer and heating 4 min at 95°C. Products were applied to 7 M urea 15% polyacrylamide (19:1 acrylamide:bisacrylamide) slab gels, electrophoresed in TBE, and analyzed by storage-phosphor scanning (Typhoon; GE Healthcare).

### Nucleotide addition in transcription initiation: de novo initiation

Reaction mixtures contained (10 μl): 0 or 10 μM SalA, 400 nM *E. coli* RNAP holoenzyme, 100 nM DNA fragment containing positions −42 to +426 of the *lacUV5(ICAP)* promoter (Naryshkin et al., 2001), 100 μM [α^32^P]ATP (0.07 Bq/fmol) in TB. Reaction components other than DNA and ATP were pre-incubated 10 min at 37°C; DNA was added and reaction mixtures were incubated 10 min at 37°C; and ATP was added and reaction mixtures were incubated 10 min at 37°C. Reactions were terminated by adding 5 μl loading buffer and heating 3 min at 95°C. Products were applied to 7 M urea 24% polyacrylamide (19:1 acrylamide:bisacrylamide) slab gels, electrophoresed in TBE, and analyzed by storage-phosphor scanning (Typhoon; GE Healthcare).

### Nucleotide addition in transcription elongation

Halted transcription elongation complexes (halted at position +29) were prepared essentially as in Revyakin et al. (2006). Reaction mixtures (13.5 μl) contained: 40 nM *E. coli* RNAP holoenzyme, 10 nM DNA fragment N25-100-tR2 (Revyakin et al., 2006), 100 μg/ml heparin, 5 μM [γ^32^P]ATP (5.5 Bq/fmol), 5 μM UTP, and 5 μM GTP in TB. Reaction components except heparin and NTPs were pre-incubated 10 min at 37°C, heparin was added and reaction mixtures were incubated 3 min at 37°C, and NTPs were added and reaction mixtures were incubated 5 min at 37°C. The resulting halted transcription elongation complexes were exposed to test compounds by addition of 0.75 μl 200 μM SalA, 0.75 μl 4 μM Rif, 0.75 μl 8 mM Stl, 0.75 μl 2 mM CBR703, 0.75 μl 400 μM MyxB, or 0.75 μl 2 mM Lpm and incubation 5 min at 37°C, and were re-started by addition of 0.75 μl 1 mM CTP and incubation 5 min at 37°C. Reactions were terminated by adding 10 μl loading buffer and heating 4 min at 95°C. Products were applied to 7 M urea 15% polyacrylamide (19:1 acrylamide:bisacrylamide) slab gels, electrophoresed in TBE, and analyzed by storage-phosphor scanning (Typhoon; GE Healthcare).

### Nucleotide addition kinetics

Reaction mixtures contained (20 µl): 0–0.4 µM SalA, 10 nM *E. coli* RNAP holoenzyme (Epicentre, Madison, WI), 5 nM DNA fragment T7A1(−65;+35) (positions −65 to +35 of the bacteriophage T7 A1 promoter [Stackhouse et al., 1989]; prepared by PCR amplification of a synthetic nontemplate-strand oligodeoxyribonucleotide), 25 μg/ml heparin, 6 mM ATP, 0–1.6 mM UTP, and 25 μM [α^32^P]CTP (0.44 Bq/fmol) in TB. Reaction components other than DNA, heparin, and NTPs were pre-incubated 30 min at 37°C. DNA was added and reaction mixtures were incubated 15 min at 37°C; heparin was added and reaction mixtures were incubated 2 min at 37°C; and NTPs were added and reactions mixtures were incubated 10 min at 37°C. Reactions were terminated by addition of 10 µl 80% formamide, 10 mM EDTA, 0.04% bromophenol blue, 0.04% xylene cyanol, and 0.08% amaranth red. Products were heated 5 min at 90°C, cooled 5 min on ice, resolved by urea-PAGE (Sambrook and Russell, 2001), and analyzed by storage-phosphor scanning (Typhoon; GE Healthcare). Data for synthesis of the trinucleotide product pppApUp[α^32^P]C were fitted to full-competitive, partial-competitive, full-noncompetitive, partial-noncompetitive, full-uncompetitive, partial-uncompetitive, full-mixed, and partial-mixed models of inhibition using the Fit-to-Model feature of the SigmaPlot Enzyme Kinetics Module v1.1 (SPSS). Fits were ranked based on the AICc statistic (Akaike Information Criterion corrected; low values best; −432.5 for full-noncompetitive model; −430.7 for partial-noncompetitive model; −429.8 for next-best model), the Sy.x statistic (standard error of the estimate; low values best; 6.647 × 10^−4^ for full-noncompetitive model; 6.651 × 10^−4^ for partial-noncompetitive model; 6.770 × 10^−4^ for next-best model), and the number of parameters (low values best; 3 for full-noncompetitive model; 4 for partial-noncompetitive model).

### Nucleotide addition in absence of RNAP trigger loop

Nucleic-acid scaffolds for assays were prepared as follows: nontemplate-strand oligodeoxyribonucleotide (5′-TCGCCAGACAGGG-3′; 0.1 μM), template-strand oligodeoxyribonucleotide (5′-CCCTGTCTGGCGATGGCGCGCCG-3′; 0.1 μM), and ^32^P-5′-end-labeled oligoribonucleotide (5′-^32^P-CGGCGCGCC-3′; 0.1 μM; 200 Bq/fmol) in 25 μl 5 mM Tris–HCl, pH 7.7, 200 mM NaCl, and 10 mM MgCl_2_, were heated 5 min at 95°C and cooled to 4°C in 2°C steps with 1 min per step using a thermal cycler (Applied Biosystems, Foster City, CA) and then were stored at −20°C.

Reaction mixtures for assays contained (10 μl): 0–64 μM SalA 40 nM wild-type or ΔTL *E. coli* RNAP core enzyme, 10 nM ^32^P-labeled nucleic-acid scaffold (200 Bq/fmol), and 20 μM ATP in TB. Reaction components except SalA and ATP were pre-incubated 5 min at 37°C, SalA was added and reaction mixtures were incubated 5 min at 37°C, and ATP was added and reaction mixtures were incubated 0.4 min (wild-type RNAP) or 10 min (ΔTL RNAP) at 37°C. Reactions were terminated by adding 10 μl loading buffer and heating 2 min at 95°C. Products were applied to 7 M urea 15% polyacrylamide (19:1 acrylamide:bisacrylamide) slab gels, electrophoresed in TBE, and analyzed by storage-phosphor scanning (Typhoon; GE Healthcare).

### Type-I and Type-II transcriptional pausing

Pausing assays were performed essentially as in Ederth et al. (2002). Reaction mixtures for formation of halted transcription elongation complexes (halted at position +29) contained (74 μl): 50 nM *E. coli* RNA polymerase holoenzyme, 40 nM DNA fragment P_T7A1_-*his*-T_*hisL*_ or P_T7A1_-*ops*_*pheP*_-T_*hisL*_ (prepared by PCR using plasmid pIA171 or pIA251 [Artsimovitch and Landick, 2000] as template, and 5′-GGAGAGACAACTTAAAGAG-3′ and 5′-CAGTTCCCTACTCTCGCATG-3′ as primers], 150 μM ApU, 1 μM [α^32^P]CTP (4 Bq/fmol), 2.5 μM ATP, 2.5 μM GTP, 20 mM Tris–HCl, pH 7.9, 20 mM NaCl, 3 mM MgCl_2_, 14 mM 2-mercaptoethanol, and 0.1 mM EDTA. Reaction components except ApU and NTPs were pre-incubated 5 min at 37°C, and ApU and NTPs were added and the reaction mixture was incubated 15 min at 37°C. The resulting halted transcription elongation complexes were exposed to SalA (or buffer blank) by addition of 4 μl 40 μM SalA (or buffer blank) and incubation for 5 min at 37°C, and were re-started by addition of 1.2 μl 10 mM ATP, 1.2 μl 10 mM CTP, 1.2 μM 10 mM UTP, 0.8 μl 10 mM GTP, and 2 μl 2 mg/ml heparin. Aliquots (10 μl) were removed at time points (0, 15, 30, 60, 120, 240, and 480 s) and after a subsequent ‘chase’ (addition of 0.1 μl 10 mM ATP, 0.1 μl 10 mM CTP, 0.15 μl 10 mM GTP, and 0.1 μl 10 mM UTP, followed by incubation 5 min at 37°C), and aliquots were combined with 10 μl loading buffer, and heated 4 min at 95°C. Products were applied to 7 M urea 15% polyacrylamide gels (19:1 acrylamide:bisacrylamide) slab gels, electrophoresed in TBE, and analyzed by storage-phosphor scanning (Typhoon; GE Healthcare). Pause half-lives and efficiencies were calculated as in Landick et al. (1996).

### Pyrophosphorolysis

Pyrophosphorolysis assays were performed essentially as in Hein et al. (2011). Nucleic-acid scaffolds were prepared by mixing 1 μM nontemplate-strand oligodeoxyribonucleotide, 1 μM template-strand oligodeoxyribonucleotide, and 0.5 μM ^32^P-5′-end-labeled oligoribonucleotide (sequences in Figure 5—figure supplement 3; RNA ^32^P-5′-end-labeled using T4 polynucleotide kinase [New England Biolabs, Ipswich, MA] and [γ^32^P]-ATP [PerkinElmer]) in 50 μl 10 mM Tris–HCl, pH 7.9, 40 mM KCl, 5 mM MgCl_2_, and then heating 2 min at 95°C, cooling to 45°C in 30 s, and cooling to 25°C in 2°C steps with 120 s per step, in a thermal cycler (Applied Biosystems). Transcription elongation complexes were reconstituted by mixing 0 or 10 μM Sal and 100 nM *E. coli* RNAP core enzyme in 90 μl 25 mM HEPES-KOH, pH 8.0, 130 mM KCl, 5 mM MgCl_2_, 1 mM DTT, 0.15 mM EDTA, 5% glycerol, and 25 μg/ml acetylated bovine serum albumin; incubating 10 min at 24°C; adding 10 µl 500 nM nucleic-acid scaffold; and incubating 15 min at 37°C. Pyrophosphorolysis was initiated by addition of 1 μl 0.05 U/μl apyrase (New England Biolabs) and 1 μl 50 mM sodium pyrophosphate; reaction mixtures were incubated at 37°C, and 10 μl aliquots were withdrawn after 0, 1, 2.5, 5, 10, 20, and 30 min and quenched by mixing with 10 μl 98% formamide, 10 mM EDTA, 0.02% bromophenol blue, and 0.02% xylene cyanol. To confirm that transcription elongation complexes were catalytically active, a ‘chase’ reaction was performed after the last time point, adding 11.4 μl reaction mixture to 0.6 μl 20 mM GTP (scaffold of panel A of Figure 5—figure supplement 3) or 0.6 μl 20 mM UTP (scaffold of panel B of Figure 5—figure supplement 3), incubating 5 min at 37°C, and withdrawing and quenching an aliquot as above. Products were applied to 7 M urea 20% polyacrylamide gels (19:1 acrylamide:bisacrylamide) slab gels, electrophoresed in TBE, and analyzed by storage-phosphor scanning (Typhoon; GE Healthcare).

### Structure determination: crystallization, crystal soaking, and cryo-cooling

Crystallization trials were performed using Crystal Former microfluidic chips (Microlytic, Burlington, MA) and SmartScreen solutions (Microlytic) (precipitant inlet: 1.5 μl screening solution; sample inlet: 1.5 μl 10 mg/ml *E. coli* RNAP holoenzyme in 10 mM Tris-HCl, pH 7.9, 100 mM NaCl, 1% glycerol; 22°C). Under one condition, small crystals appeared within 2 days. Conditions were optimized using the hanging-drop vapor-diffusion technique at 22°C. The optimized conditions (reservoir: 500 μl 0.1 M HEPES, pH 7.0, 0.2 M CaCl_2_, and 18% PEG400; drop: 1 μl 10 mg/ml *E. coli* RNAP holoenzyme in 10 mM Tris-HCl, pH 7.9, 100 mM NaCl, 1% glycerol plus 1 μl reservoir solution; 22°C) yielded large crystals with dimensions of 0.2 mm × 0.2 mm × 0.2 mm in one week. SalA and Sal–Br were soaked into RNAP crystals, yielding RNAP–Sal and RNAP–Sal–Br crystals, by addition of 0.2 μl 20 mM SalA or Sal–Br in (±)-2-methyl-2,4-pentanediol (Hampton Research, Aliso Viejo, CA) to the crystallization drop and incubation 30 min at 22°C. RNAP, RNAP-Sal, and RNAP–Sal–Br crystals were transferred to reservoir solutions containing 15% (vol/vol) (2R, 3R)-(−)-2, 3-butanediol (Sigma–Aldrich) and then flash-cooled with liquid nitrogen.

### Structure determination: data collection and reduction

Diffraction data were collected from cryo-cooled crystals at Cornell High Energy Synchrotron Source beamline F1 and at Brookhaven National Laboratory beamline X25. Data were processed using HKL2000 (Otwinowski and Minor, 1997).

### Structure determination: structure solution and refinement

The structure of *E. coli* RNAP holoenzyme was solved by molecular replacement using AutoMR (McCoy et al., 2007; Adams et al., 2010). The search model was generated by starting with the crystal structure of *T. thermophilus* RNAP-promoter open complex (PDB 4G7H; Zhang et al., 2012), deleting DNA and non-conserved protein domains, modelling *E. coli* α^I^ and α^II^ subunit N-terminal domains by superimposing the crystal structure of *E. coli* α N-terminal domain dimer (PDB 1BDF; Zhang and Darst, 1998), and modelling *E. coli* β, β', ω, and σ^70^ subunits using Sculptor (Bunkóczi and Read, 2011; backbone and sidechain atoms for identical residues; backbone and Cβ atoms for non-identical residues). Two RNAP molecules are present in the asymmetric unit. Crystal structures of *E. coli* α subunit C-terminal domain (PDB 3K4G; Lara-González et al., 2010), the *E. coli* β subunit β2-βi4 and βflap-βi9 domains (PDB 3LTI and PDB 3LU0; Opalka et al., 2010), and *E. coli* σ^70^ region 2 (PDB 1SIG; Malhotra et al., 1996) were fitted manually to the (Fo-Fc) difference electron density map. Early-stage refinement of the structure was performed using Phenix (Adams et al., 2010) and included rigid-body refinement of each RNAP molecule in the asymmetric unit, followed by rigid-body refinement of each subunit of each RNAP molecule, followed by rigid-body refinement of 216 segments of each RNAP molecule, followed by group B-factor refinement with one B-factor group per residue, using Phenix (methods as in Zhang et al., 2012). Density modification, including non-crystallographic-symmetry averaging and solvent flattening using a locally modified version of DM (Collaborative Computational Project, 1994), was performed to remove model bias and to improve phases. The resulting maps allowed segments that were not present in the search model to be built manually using Coot (Emsley et al., 2010). Cycles of iterative model building with Coot and refinement with Phenix improved the model. The final *E. coli* RNAP holoenzyme model, refined to Rwork and Rfree of 0.276 and 0.325, respectively, has been deposited in the PDB with accession code 4MEY (Supplementary file 2).

The structures of the *E. coli* RNAP–SalA and RNAP–Sal–Br complexes were solved by molecular replacement in AutoMR, using the above crystal structure of *E. coli* RNAP holoenzyme as the search model. For each structure, after rigid-body refinement with 216 domains, an electron density feature corresponding to one molecule of SalA per holoenzyme was clearly visible in the (Fo-Fc) difference map. A structural model of SalA derived from the crystal structure of SalB (CSD 50962; Trischman et al., 1994; enantiomorph corrected based on Moore et al., 1999) was fitted to the (Fo-Fc) difference map with minor adjustments of SalA conformation, and the fit was confirmed by the position of the peak of Br anomalous difference density for the RNAP–Sal–Br complex. The final *E. coli* RNAP–SalA complex model, refined to Rwork and Rfree of 0.286 and 0.325, respectively, has been deposited in the PDB with accession code 4MEX (Supplementary file 2).

## Additional files

Supplementary file 1. Plasmid-borne induced Sal-resistant mutants: sequences and properties. **DOI:** http://dx.doi.org/10.7554/eLife.02451.017

Supplementary file 2. Crystal structures of *E. coli* RNAP holoenzyme and *E. coli* RNAP holoenzyme in complex with Sal: crystallization and refinement statistics. **DOI:** http://dx.doi.org/10.7554/eLife.02451.018

Supplementary file 3. Crystal structure of *E. coli* RNAP holoenzyme in complex with a bromine-containing Sal derivative: crystallization and refinement statistics. **DOI:** http://dx.doi.org/10.7554/eLife.02451.019

### Major datasets

The following datasets were generated:

FengY, ZhangY, ArnoldE, EbrightRH, 2013, Crystal structure of *Escherichia coli* RNA polymerase holoenzyme, http://www.rcsb.org/pdb/search/structidSearch.do?structureId=4MEY, Publicly available at RCSB Protein Data Bank.

FengY, ZhangY, ArnoldE, EbrightRH, 2013, Crystal structure of *Escherichia coli* RNA polymerase in complex with salinamide A, http://www.rcsb.org/pdb/search/structidSearch.do?structureId=4MEX, Publicly available at RCSB Protein Data Bank. http://www.wwpdb.org/policy.html.

The following previously published datasets were used:

ZhangY, FengY, ChatterjeeS, TuskeS, HoMX, ArnoldE, EbrightRH, 2012, Crystal structure of *Thermus thermophilus* transcription initiation complex, http://www.pdb.org/pdb/explore/explore.do?structureId=4g7h, Publicly available at RCSB Protein Data Bank.

Lara-GonzalezS, BirktoftJJ, LawsonCL, 2010, Crystal structure of *E. coli* RNA polymerase alpha subunit C-terminal domain, http://www.pdb.org/pdb/explore/explore.do?structureId=3k4g, Publicly available at RCSB Protein Data Bank.

OpalkaN, BrownJ, LaneWJ, TwistKA, LandickR, AsturiasFJ, DarstSA, 2010, Crystal structure of the *Escherichia coli* RNA polymerase beta subunit beta2-betai4 domains, http://www.pdb.org/pdb/explore/explore.do?structureId=3lti, Publicly available at RCSB Protein Data Bank.

OpalkaN, BrownJ, LaneWJ, TwistKA, LandickR, AsturiasFJ, DarstSA, 2010, Molecular model of *Escherichia coli* core RNA polymerase, http://www.pdb.org/pdb/explore/explore.do?structureId=3lu0, Publicly available at RCSB Protein Data Bank.

MalhotraA, SeverinovaE, DarstSA, 1996, Crystal structure of a sigma70 subunit fragment from *Escherichia coli* RNA polymerase, http://www.pdb.org/pdb/explore/explore.do?structureId=1sig, Publicly available at RCSB Protein Data Bank.

VassylyevDG, SekineS, LaptenkoO, LeeJ, VassylyevaMN, BorukhovS, YokoyamaS, 2002, Crystal structure of the RNA polymerase holoenzyme from *Thermus thermophilus* at 2.6A resolution, http://www.pdb.org/pdb/explore/explore.do?structureId=1iw7, Publicly available at RCSB Protein Data Bank.

VassylyevDG, VassylyevaMN, ZhangJ, PalangatM, ArtsimovitchI, LandickR, 2007, Crystal structure of the *T. thermophilus* RNAP polymerase elongation complex with the NTP substrate analog, http://www.pdb.org/pdb/explore/explore.do?structureId=2o5j, Publicly available at RCSB Protein Data Bank.

WeixlbaumerA, LeonK, LandickR, DarstSA, 2013, Crystal structures of bacterial RNA Polymerase paused elongation complexes, http://www.pdb.org/pdb/explore/explore.do?structureId=4gzy, Publicly available at RCSB Protein Data Bank.

TuskeS, SarafianosSG, WangX, HudsonB, SinevaE, MukhopadhyayJ, BirktoftJJ, LeroyO, IsmailS, ClarkAD, DhariaC, NapoliA, LaptenkoO, LeeJ, BorukhovS, EbrightRH, ArnoldE, 2005, Structure of *Thermus thermophilus* RNA polymerase holoenzyme in complex with the antibiotic streptolydigin, http://www.pdb.org/pdb/explore/explore.do?structureId=1zyr, Publicly available at RCSB Protein Data Bank.

**Reporting standards:** Standard used to collect data.
