# Supplementary material for: Transcription inhibition by the depsipeptide antibiotic salinamide A
Source: eLife. 2014 Apr 30;3:e02451. doi: 10.7554/eLife.02451 (PMC4029172; doi:10.7554/eLife.02451)
Supplement: Supplementary file 1. — Plasmid-borne induced Sal-resistant mutants: sequences and properties. DOI: http://dx.doi.org/10.7554/eLife.02451.017 [file elife02451s001.doc]

**Supplementary file 1. Plasmid-borne induced Sal-resistant mutants:**

**sequences and properties.**

| **amino acid**  **substitution** | **number of**  **independent isolates** | **resistance level (MIC/MIC_wild-type_)^a^** | | **ability to complement *rpoC*^ts^ or *rpoB*^ts^** |
| --- | --- | --- | --- | --- |
|  |  | **SalA** | **SalB** |  |
|  |  |  |  |  |
| ***rpoC* (RNAP β′ subunit)** |  |  |  |  |
|  |  |  |  |  |
| 504 Gln→Pro | 1 | 2 | 1 | + |
| 735 Ala→Thr | 1 | 2 | 2 | + |
| 758^b^ Pro→Ser | 1 | 4 | 2 | + |
| 758^b^ Pro→Thr | 1 | 4 | 2 | + |
| 780^b^ Arg→Cys | 1 | 16 | 8 | + |
| 782^b^ Gly→Cys | 1 | 2 | 2 | + |
|  |  |  |  |  |
| ***rpoB* (RNAP β subunit)** |  |  |  |  |
|  |  |  |  |  |
| 561 Ile→Ser | 2 | 2 | 2 | + |
| 665 Ala→Glu | 1 | 4 | 4 | + |
| 680 Leu→Met | 1 | 2 | 2 | + |
|  |  |  |  |  |

^a^ MIC_wild-type,SalA_ = 0.049 µg/ml; MIC_wild-type_,SalB = 0.20 µg/ml.

^b^ Spontaneous Sal-resistant mutants isolated at same residue (see Figure 2C).
