# Supplementary material for: Transcription inhibition by the depsipeptide antibiotic salinamide A
Source: eLife. 2014 Apr 30;3:e02451. doi: 10.7554/eLife.02451 (PMC4029172; doi:10.7554/eLife.02451)
Supplement: Supplementary file 2. — Crystal structures of E. coli RNAP holoenzyme and E. coli RNAP holoenzyme in complex with Sal: crystallization and refinement statistics. DOI: http://dx.doi.org/10.7554/eLife.02451.018 [file elife02451s002.doc]

**Supplementary file 2. Crystal structures of *E. coli* RNAP holoenzyme and *E. coli* RNAP holoenzyme in complex with Sal: crystallization and refinement statistics.**

|  |  |  |
| --- | --- | --- |
| dataset | Eco RNAP | Eco RNAP-Sal |
| beamline | CHESS-F1 | BNL-X25 |
| space group | P2_1_2_1_2_1_ | P2_1_2_1_2_1_ |
| resolution range | 50.00-3.90 Å (3.97-3.90 Å) | 50.00-3.90 Å (3.97-3.90 Å) |
| cell parameters (Å, °) | a=186.4, b=207.2, c=308.3  α=90.0, β=90.0, γ=90.0 | a=185.8, b=208.2, c=308.2  α=90.0, β=90.0, γ=90.0 |
| completeness | 0.997 (0.996) | 0.998 (0.997) |
| multiplicity | 5.9 (4.8) | 6.4 (4.9) |
| mean I/σ | 13.2 (1.8) | 15.5 (1.5) |
| Rmerge | 0.115 (0.625) | 0.118 (0.891) |
| Rwork | 0.276 | 0.286 |
| Rfree | 0.325 | 0.325 |
| bond-length rmsd | 0.004 Å | 0.004 Å |
| bond-angle rmsd | 0.884° | 1.009° |
| PDB code | 4MEY | 4MEX |
|  |  |  |
