# Supplementary material for: Transcription inhibition by the depsipeptide antibiotic salinamide A
Source: eLife. 2014 Apr 30;3:e02451. doi: 10.7554/eLife.02451 (PMC4029172; doi:10.7554/eLife.02451)
Supplement: Supplementary file 3. — Crystal structure of E. coli RNAP holoenzyme in complex with a bromine-containing Sal derivative: crystallization and refinement statistics. DOI: http://dx.doi.org/10.7554/eLife.02451.019 [file elife02451s003.doc]

**Supplementary file 3. Crystal structure of *E. coli* RNAP holoenzyme in complex with a bromine‑containing Sal derivative: crystallization and refinement statistics.**

|  |  |
| --- | --- |
| dataset | Eco RNAP-Sal- Br |
| beamline | CHESS-F1 |
| space group | P2_1_2_1_2_1_ |
| resolution range | 50.00-4.70 Å (4.78-4.70 Å) |
| cell parameters (Å, °) | a=186.2, b=208.2, c=308.2  α=90.0, β=90.0, γ=90.0 |
| completeness | 1.000 (1.000) |
| multiplicity | 16.6 (16.9) |
| mean I/σ | 17.3 (3.4) |
| Rmerge | 0.200 (0.962) |
|  |  |
